# Supplementary material for: Seroepidemiological survey and seropositivity rate for Trypanosoma cruzi infection in a community-based cardiac screening initiative in Feira de Santana, Bahia, Brazil
Source: PLoS Negl Trop Dis. 2026 Jan 2;20(1):e0013892. doi: 10.1371/journal.pntd.0013892 (PMC12782403; doi:10.1371/journal.pntd.0013892)
Supplement: Attachment — Submitted filename: PNTD-D-25-00569_reviewer.pdf [file pntd.0013892.s002.pdf]

PLOS Neglected Tropical Diseases

Seroepidemiological survey and seropositivity rate for Trypanosoma cruzi infection in a community-based cardiac screening initiative in Feira de Santana, Bahia, Brazil

--Manuscript Draft--

**Manuscript Number:**	PNTD-D-25-00569
**Full Title:**	Seroepidemiological survey and seropositivity rate for Trypanosoma cruzi infection in a community-based cardiac screening initiative in Feira de Santana, Bahia, Brazil
**Short Title:**	Seropositivity rate of T. cruzi infection in cardiac screening in Bahia
**Article Type:**	Research Article
**Keywords:**	Chagas disease; Trypanosoma cruzi; seropositivity rate; risk factors; cardiac screening; vector control
**Abstract:**	Chagas disease (CD), caused by Trypanosoma cruzi, is a significant public health issue in Latin America, particularly in endemic regions. This study integrates a seroepidemiological survey with large-scale echocardiographic screening to estimate the prevalence of T. cruzi infection and identify associated risk factors. Peripheral blood samples were analyzed using in-house ELISA with IBMP chimeric antigens. Among 1,115 participants, 140 were selected for serological test based on clinical data and an ECG-AI-derived algorithm with cases and controls matched in a 2:1 ratio. Of these, 8.5% tested seropositive, with household exposure to triatomines identified as the strongest risk factor (prevalence ratio = 4.38, p = 0.004). Most seropositive individuals were migrants from other endemic areas, underscoring the influence of population mobility on CD epidemiology. This study highlights the importance of integrating diagnostic tools and vector control strategies into community-based health initiatives to improve early detection, reduce disease burden, and inform public health interventions in underserved regions.
**Additional Information:**	
**Question**	**Response**
**Government Employee**<br><br>Are you or any of the contributing authors an employee of the United States government?<br><br>Manuscripts authored by one or more US Government employees are not copyrighted, but are licensed under a CC0 Public Domain Dedication, which allows unlimited distribution and reuse of the article for any lawful purpose. This is a legal requirement for US Government employees.<br><br>This will be typeset if the manuscript is accepted for publication.	No - No authors are employees of the U.S. government.
**Financial Disclosure**<br><br>Enter a financial disclosure statement that describes the sources of funding for the	Yes

Powered by Editorial Manager® and ProduXion Manager® from Aries Systems Corporation

work included in this submission. Review the submission guidelines for detailed requirements. View published research articles from *PLOS Neglected Tropical Diseases* for specific examples.<br><br>This statement is required for submission and **will appear in the published article** if the submission is accepted. Please make sure it is accurate.<br><br>**Funded studies**<br>Enter a statement with the following details:<br>• Initials of the authors who received each award<br>• Grant numbers awarded to each author<br>• The full name of each funder<br>• URL of each funder website<br>• Did the sponsors or funders play any role in the study design, data collection and analysis, decision to publish, or preparation of the manuscript?<br><br>Did you receive funding for this work?	
Please add funding details.<br>as follow-up to "**Financial Disclosure**<br><br>Enter a financial disclosure statement that describes the sources of funding for the work included in this submission. Review the submission guidelines for detailed requirements. View published research articles from *PLOS Neglected Tropical Diseases* for specific examples.<br><br>This statement is required for submission and **will appear in the published article** if the submission is accepted. Please make sure it is accurate.	The author(s) declare that financial support was received for the research, authorship, and/or publication of this article. This study was supported by the Edwards Lifesciences Foundation and the Coordination for the Improvement of Higher Education Personnel in Brazil (CAPES; Finance Code 001). NITZ and FLNS are research fellows of the National Council for Scientific and Technological Development-Brazil (CNPq; processes 304894/2023-0 and 306448/2023-8, respectively). The funders had no involvement in the study design, data collection and analysis, decision to publish, or preparation of the manuscript.

Powered by Editorial Manager® and ProduXion Manager® from Aries Systems Corporation

**Funded studies**<br>Enter a statement with the following details:<br>• Initials of the authors who received each award<br>• Grant numbers awarded to each author<br>• The full name of each funder<br>• URL of each funder website<br>• Did the sponsors or funders play any role in the study design, data collection and analysis, decision to publish, or preparation of the manuscript?<br><br>Did you receive funding for this work?"	
Please select the country of your main research funder (please select carefully as in some cases this is used in fee calculation).<br>as follow-up to "**Financial Disclosure**<br><br>Enter a financial disclosure statement that describes the sources of funding for the work included in this submission. Review the submission guidelines for detailed requirements. View published research articles from *PLOS Neglected Tropical Diseases* for specific examples.<br><br>This statement is required for submission and **will appear in the published article** if the submission is accepted. Please make sure it is accurate.<br><br>**Funded studies**<br>Enter a statement with the following details:<br>• Initials of the authors who received each award<br>• Grant numbers awarded to each author<br>• The full name of each funder<br>• URL of each funder website<br>• Did the sponsors or funders play any role in the study design, data collection and analysis, decision to publish, or preparation of the manuscript?<br><br>Did you receive funding for this work?"	BRAZIL - BR
**Competing Interests**	The authors declare that the research was conducted in the absence of any

On behalf of all authors, disclose any competing interests that could be perceived to bias this work.<br><br>This statement will be typeset if the manuscript is accepted for publication.<br><br>Review the instructions link below and PLOS NTDs' competing interests policy to determine what information must be disclosed at submission.	commercial or financial relationships that could be construed as a potential conflict of interest.
**Data Availability**<br><br>Provide a **Data Availability Statement** in the box below. This statement should detail where the data used in this submission can be accessed. This statement will be typeset if the manuscript is accepted for publication.<br><br>Before publication, authors are required to make all data underlying their findings fully available, without restriction. Review our PLOS Data Policy page for detailed information on this policy. Instructions for writing your Data Availability statement can be accessed via the Instructions link below.	The original contributions presented in the study are included in the article/supplementary material, further inquiries can be directed to the corresponding author.

Powered by Editorial Manager® and ProduXion Manager® from Aries Systems Corporation

Manuscript Click here to access/download;Manuscript;Main document.docx

Seroepidemiological survey and seropositivity rate for *Trypanosoma cruzi* infection in a community-based cardiac screening initiative in Feira de Santana, Bahia, Brazil

Running title: Seropositivity rate of *T. cruzi* infection in cardiac screening in Bahia

Felipe Silva Santos de Jesus[1,2¶], Isabella Moreira Gonzalez Fonseca[3¶], Ângelo Antônio Oliveira Silva[1,2], Noilson Lázaro Sousa Gonçalves[1], Daniel Dias Sampaio[2], Deborah Bittencourt Mothé Fraga[4], Paola Alejandra Fiorani Celedon[5], Dalila Luciola Zanette[5], Nilson Ivo Tonin Zanchin[6], Antônio Luiz Pinho Ribeiro[3], Maria Carmo Pereira Nunes[3], Manoel Otávio da Costa Rocha[3], Craig Sable[7], Fred Luciano Neves Santos[1,2,8*]

[1] Advanced Health Public Laboratory, Gonçalo Moniz Institute, Oswaldo Cruz Foundation, Salvador, Bahia, Brazil

[2] Interdisciplinary Research Group in Biotechnology and Epidemiology of Infectious Diseases (GRUPIBE), Gonçalo Moniz Institute, Oswaldo Cruz Foundation, Salvador, Bahia, Brazil

[3] Medical School and University Hospital, Federal University from Minas Gerais, Belo Horizonte, Minas Gerais, Brazil

[4]Laboratory of Interaction Parasite Host and Epidemiology (LAIPHE), Gonçalo Moniz Institute, Oswaldo Cruz Foundation, Salvador, Bahia, Brazil

[5]Laboratory for Applied Science and Technology in Health, Carlos Chagas Institute, Oswaldo Cruz Foundation, Curitiba, Paraná, Brazil

[6]Structural Biology and Protein Engineering Laboratory, Carlos Chagas Institute, Oswaldo Cruz Foundation, Curitiba, Paraná, Brazil

[7]Ochsner Children's Hospital, New Orleans, Louisiana, United States

[8] Integrated Translational Program in Chagas disease from Fiocruz – Fio-Chagas, Rio de Janeiro, Rio de Janeiro, Brazil.

* Corresponding author:

E-mail: fred.santos@fiocruz.br (FNLS)

¶These authors contributed equally to this work.

Abstract

Chagas disease (CD), caused by *Trypanosoma cruzi*, is a significant public health issue in Latin America, particularly in endemic regions. This study integrates a seroepidemiological survey with large-scale echocardiographic screening to estimate the prevalence of *T. cruzi* infection and identify associated risk factors. Peripheral blood samples were analyzed using in-house ELISA with IBMP chimeric antigens. Among 1,115 participants, 140 were selected for serological test based on clinical data and an ECG-AI-derived algorithm with cases and controls matched in a 2:1 ratio. Of these, 8.5% tested seropositive, with household exposure to triatomines identified as the strongest risk factor (prevalence ratio = 4.38, p = 0.004). Most seropositive individuals were migrants from other endemic areas, underscoring the influence of population mobility on CD epidemiology. This study highlights the importance of integrating diagnostic tools and vector control strategies into community-based health initiatives to improve early detection, reduce disease burden, and inform public health interventions in underserved regions.

Keywords: Chagas disease, *Trypanosoma cruzi*, seropositivity rate, risk factors, cardiac screening, vector control.

Author Summary

Chagas disease, caused by the parasite *Trypanosoma cruzi*, remains a major health concern in Latin America, especially in areas with limited access to healthcare. In this study, we combined artificial intelligence tools and cardiac screening with serological testing to detect undiagnosed infections in Feira de Santana, a city in the state of Bahia,

Brazil. By analyzing electrocardiogram data and environmental risk factors, we identified individuals at risk for Chagas disease and confirmed infection using advanced laboratory tests. Our results showed that people who reported seeing triatomine bugs inside their homes were much more likely to be infected, emphasizing the role of vector exposure in disease transmission. We also found that most infected individuals were unaware of their condition and had migrated from other endemic regions. These findings highlight the importance of combining innovative technologies with community-based screening to improve early diagnosis and guide public health strategies in vulnerable populations.

Introduction

Chagas disease (CD), caused by the protozoan *Trypanosoma cruzi*, is a significant public health issue in Latin America, affecting millions and contributing to considerable morbidity and mortality [1,2]. Despite advances in control measures, gaps in early detection and treatment persist, particularly in underserved regions [3]. Chronic CD can lead to cardiac involvement, which represents the leading cause of mortality, highlighting the need for innovative strategies to improve healthcare delivery and patient outcomes.

Feira de Santana, Bahia, is a highly CD endemic region in Brazil with a substantial burden, where Chagas-related mortality significantly impacts local health [4]. Socioeconomic disparities and inadequate healthcare infrastructure exacerbate diagnostic and treatment delays. International initiatives, such as the *Every Heartbeat Matters Program* [5], have emerged as promising approaches to address these challenges. This initiative integrates advanced diagnostic technologies, including telehealth-enabled electrocardiography (teleECG) and targeted echocardiographic screening, to enhance the detection and management of structural heart disease in underserved populations.

This study leverages data from a seroepidemiological survey conducted alongside the *Every Heartbeat Matters* initiative in Feira de Santana. The primary objectives were to estimate the seropositivity rate of *T. cruzi* infection, identify associated risk factors, and assess the feasibility of integrating seroepidemiological surveys with echocardiographic screening programs in endemic settings. The findings aim to inform public health strategies and strengthen the evidence base for effective CD management in high-burden areas.

Methods

Study population

This cross-sectional study included 1,115 adults who were referred for a large-scale screening, which comprised a structured questionnaire, electrocardiogram (ECG), and echocardiography. An artificial intelligence (AI)-based algorithm was implemented to identify individuals at risk for CD by integrating data from 12-lead ECGs with epidemiological variables to estimate the probability of infection.

The risk classification system was based on a combination of three predefined epidemiological questions and the AI-ECG-derived score, categorizing individuals into low, medium, and high risk for CD. The three selected questions assessed whether participants: (1) had a family member diagnosed with CD, (2) had lived in a region with known triatomine (kissing bug) exposure, and (3) had resided in a wooden house, a known risk factor for vector transmission.

A combination of clinical data and AI-ECG findings was used to generate a risk score, flagging individuals as positive cases. Those identified as positive underwent serological

test for CD. For comparison, controls were randomly selected at a 2:1 ratio (controls to cases) and underwent CD serological testing.

Specimen Collection and Analysis

Peripheral blood samples (9 mL) were collected in clot-activator tubes with gel separators (Vacuplast, Brazil). Following a 30-minute clotting period at room temperature, samples were centrifuged at 3,000 × g for 10 minutes. Serum was aliquoted into cryotubes, transported to the Gonçalo Moniz Institute, and stored at −20 °C until analysis.

Serological Testing

Anti-*T. cruzi* antibodies were detected using an in-house ELISA based on IBMP chimeric antigens (IBMP-8.1, IBMP-8.2, IBMP-8.3, and IBMP-8.4), following established protocols [6]. Recombinant antigens were expressed in *Escherichia coli* BL21-Star (DE3) and purified via affinity and ion-exchange chromatography. Protein concentrations were quantified fluorometrically using a Qubit 2.0 fluorometer (Invitrogen, Carlsbad, CA, USA). Immunoassays were performed using transparent 96-well flat-bottom microplates (Greiner Bio-One, Austria). The plates were sensitized with the antigens and blocked with a solution of Well-Champion (Ken-Em-Tec Diagnostics A/S, Denmark). Serum samples were diluted 1:100 in PBS-T (PBS containing 0.05% Tween-20; pH 7.4) and incubated at 37 °C for 30 minutes. After washing with PBS-T, a goat anti-human IgG-HRP conjugate (Bio-Manguinhos, FIOCRUZ, Brazil) was added and incubated at 37 °C for 30 minutes. Following additional washing, immune complexes were identified using TBM substrate (Kem-En-Tec, Denmark) with a 10-minute incubation in the dark at room temperature. The reaction was stopped with 0.3 M H_2SO_4,

and optical density (OD) was measured at 450 nm using a SPECTRAmax 340PC microplate reader (Molecular Devices, USA).

Reference Testing

Latent class analysis (LCA) was employed to classify *T. cruzi* infection status in the absence of a gold standard for chronic CD diagnosis [7–9]. Seropositivity was defined as reactivity in at least two of four ELISAs, with posterior probabilities ranging from 87.9% to 100%. Seronegativity was defined as nonreactivity in at least three ELISAs, with probabilities ranging from 0% to 0.8%. Positive samples were validated using commercial assays, including Gold ELISA Chagas® (REM Indústria e Comércio Ltda, Brazil) and Bioelisa Chagas Recombinante® (Bioclin Quibasa Química Básica, Brazil).

Ethics

This study adhered to the principles of the Declaration of Helsinki and was approved by the Institutional Review Board (IRB) of the Gonçalo Moniz Institute, Oswaldo Cruz Foundation (IGM-FIOCRUZ), Salvador, Bahia, Brazil (protocol no. 67809417.0.0000.0040). Written informed consent was obtained from all participants before enrollment.

Data Analysis

Descriptive statistics were used to summarize the demographic and epidemiological characteristics of the study population. Age was reported as the median with the interquartile range (IQR), while categorical variables (e.g., sex, serological test results) were expressed as absolute and relative frequencies. Associations between *T. cruzi*

seropositivity and potential risk factors, including prior exposure to triatomines, contact with kissing bugs, presence of a chicken coop, and familial history of CD, were evaluated using contingency tables. Seropositivity rates were calculated as the proportion of seropositive cases among exposed individuals relative to non-exposed individuals, with 95% confidence intervals (CI) and p-values derived from Fisher's exact test. Statistical significance was set at p-value < 0.05. Additionally, prevalence ratio (PR) was performed to assess the association of sociodemographic and environmental factors with positivity. Multiple logistic regression integrated data from 12-lead ECGs plus epidemiological variables to calculate the probability of CD.

Results

Between August 24 and 27, 2024, 1,115 individuals participated in the *Every Heartbeat Matters* program, a large-scale ECG and echocardiographic screening, at the Holy House of Mercy in Feira da Santana (Fig 1). In the entire screened population, the median age was 57 years (IQR: 44–64 years), 78.4% were women with a female-to-male ratio of 4.9:1. A total of 284 individuals (27.5%) presented minor ECG abnormalities, while 118 (11.4%) had major abnormalities.

Among the selected participants, the AI algorithm identified 121 individuals for serological testing, including 80 classified as high risk for CD and 41 individuals randomly chosen as controls based on predefined criteria, while 994 were excluded. Of the 73 participants with cardiac abnormalities who underwent blood collection for CD diagnosis, 13 (17.8%) tested positive for *T. cruzi*. In contrast, all 39 control subjects who underwent blood collection tested negative, resulting in a seropositivity rate of 0%. The

overall seropositivity rate of *T. cruzi* infection among all tested participants was 11.6% (13/112) (Fig 1).

Among individuals with false-positive AI alerts, ECG abnormalities were observed in six cases classified as major and 16 as minor. Regarding screening echocardiography, only two participants with confirmed CD exhibited left or right ventricular involvement, while 18 had entirely normal echocardiographic findings.

Fig 1. Study design following the Standards for Reporting of Diagnostic Accuracy Studies (STARD) guidelines.

Latent class analysis classified 99 samples (88.4%) as *T. cruzi*-negative, demonstrating no reactivity against the four IBMP antigens, with a posterior probability of zero for positivity. Thirteen samples (11.6%) were classified as *T. cruzi*-positive. Among these, 61.5% (n = 8) reacted with all four IBMP antigens, 15.4% (n = 2) with three antigens, and 23.1% (n = 3) with two antigens. Posterior probabilities for positivity ranged from 96.9% to 100%, confirming their classification as *T. cruzi*-positive, with Gold ELISA Chagas® and Bioelisa Chagas Recombinante® detecting seropositivity in 100% (13/13) of cases. These results highlight the strong concordance between IBMP chimeric antigen-based ELISA and commercial assays. Table 1 summarizes the serological findings, detailing reactivity patterns across IBMP ELISA assays and commercial kits.

Table 1. Reactivity patterns and classification of *Trypanosoma cruzi* seropositivity.

Participant number	In-house assays with IBMP antigens						Commercial kits	
	**-8.1**	**-8.2**	**-8.3**	**-8.4**	**LCA**	**P (%)***	**GEC[1]**	**BIO[2]**
1	2.16	0.55	1.07	0.41	POS	96.9	2.64	3.96
2	2.00	0.59	1.03	0.58	POS	96.9	2.54	3.53
3	2.04	1.58	1.83	2.17	POS	100	12.41	3.03
4	0.75	1.47	1.53	1.54	POS	100	7.35	3.22
5	2.75	1.84	1.72	1.85	POS	100	9.54	2.57
6	2.95	1.83	1.69	2.47	POS	100	7.89	3.15
7	2.41	1.54	1.85	1.49	POS	100	11.01	2.99
8	1.44	0.62	0.58	1.01	POS	99	2.47	2.68
9	1.95	1.17	0.76	1.23	POS	100	5.27	2.29
10	1.89	1.77	1.82	2.48	POS	100	12.71	3.57
11	2.84	1.08	1.90	2.03	POS	100	10.56	3.26
12	2.56	1.52	1.81	1.73	POS	100	10.66	2.33
13	2.24	1.16	1.44	1.75	POS	100	9.01	2.61

[1]GEC (Gold ELISA Chagas®); [2]BIO (Bioelisa Chagas Recombinante®); LCA (Latent Class Analysis); NP (Not Performed); POS (Positive). *Probability of being *T. cruzi*-positive by LCA.

Among *T. cruzi*-positive individuals, the median age was 60 years (IQR: 39–69 years), with a female-to-male ratio of 5.5:1, while *T. cruzi*-negative individuals had a median age of 56 years (IQR: 44–64 years) and a female-to-male ratio of 4.8:1. Notably, 69.2% of

seropositive individuals were not born in Feira de Santana (Fig 2), having migrated from other endemic areas in Bahia, including Conceição do Jacuípe (n = 1), Coração de Maria (n = 1), Ipirá (n = 1), Mundo Novo (n = 1), Muniz Ferreira (n = 1), Santa Maria da Vitória (n = 1), Utinga (n = 1), and Santo Estevão (n = 2). This suggests migration as a potential factor influencing local epidemiological trends.

Although most participants were aware of their clinical condition, 23% of *T. cruzi*-positive individuals were unaware of their infection status (Fig 2), highlighting gaps in diagnosis and awareness.

Fig 2. Geographic origins of *Trypanosoma cruzi*-positive individuals in Bahia, Brazil. Public domain digital maps were obtained from the Brazilian Institute of Geography and Statistics (IBGE) cartographic database in shapefile format (.shp), which was subsequently reformatted and analyzed using QGIS version 3.10 (Geographic Information System, Open-Source Geospatial Foundation Project. http://qgis.osgeo.org).

All *T. cruzi*-positive individuals were older than 46 years and were either retired or beneficiaries of government assistance programs (Fig 2), except for one family cluster. This family, comprising parents and three children, likely contracted the infection through oral transmission after consuming açaí juice in February 2024 near Feira de Santana. At the time of exposure, the parents were 40 and 38 years old. Among the children, a 13-year-old girl and a 9-year-old boy tested positive, while a third child who did not consume the juice tested negative. The infected family members received etiological treatment and remained under clinical supervision.

In univariate analysis, household observation of triatomine bugs was significantly associated with *T. cruzi* seropositivity (PR = 4.38, 95% CI: 1.44–13.3, p = 0.004). Other variables, including general knowledge about the vector, the presence of a chicken coop near the house, and a family history of CD, were not significantly associated with seropositivity. This result indicates that individuals who reported seeing these insects indoors had an almost fourfold increase in the likelihood of infection (Table 2).

Table 2. Univariate Analysis of Factors Associated with *Trypanosoma cruzi* Seropositivity.

Variables	Univariate analysis		
	PR	95%CI	p-value
Prior exposure to triatomines	4.38	1.44 to 13.3	0.004
Presence of a chicken coop	0.82	0.27 to 2.48	0.730
Familial history of CD	1.81	0.59 to 5.55	0.285

PR (Prevalence ratio), β (Log-odds coefficient), CD (Chagas disease) CI (Confidence interval).

Discussion

This study was designed to enhance diagnostic strategies for CD by leveraging large-scale digital health AI tools to overcome barriers in identifying patients with CD, yielding noteworthy findings. A significant association was identified between the household observation of triatomine bugs and *T. cruzi* seropositivity, underscoring vector exposure as a key factor for CD occurrence in endemic regions. While other environmental factors such as the presence of chicken coops near houses and familial history of CD were not

relevant, direct exposure to the vector emerged as the strongest factor associated with higher prevalence of infection. These findings are consistent with previous studies emphasizing the critical role of domestic vector infestation in sustaining transmission cycles [10,11].

The overall seropositivity rate of *T. cruzi* infection in this study (8.5%) is comparable to reports from other endemic areas in Bahia [12–14], reinforcing the persistent public health challenge posed by CD. The high seropositivity rate among individuals reporting the triatomine presence in their households highlights the need for effective vector control programs. In addition to reducing domestic infestation, community education on vector recognition and preventive measures is essential to disrupt transmission dynamics.

A notable observation was that most *T. cruzi*-positive individuals currently residing in Feira de Santana were born in other endemic areas. This suggests that many infections likely occurred before migration, highlighting the impact of population mobility on CD epidemiology. However, it remains unclear whether migration itself serves as an independent risk factor for *T. cruzi* transmission or if these individuals were exposed to their hometowns before relocating. The absence of longitudinal data on the timing of infection limits causal inferences related to migration, as transmission could have occurred before or after resettlement. Furthermore, variations in vector control programs and healthcare access between endemic regions may influence infection risks differently across migrant populations. These findings present efforts to design targeted interventions and emphasize the importance of integrating migrant populations into local healthcare systems. Implementing tailored approaches that improve access to diagnostic and treatment services for migrant communities are essential to reduce the overall burden of CD in endemic regions.

Analysis revealed that individuals who reported finding triatomines in their households presented four times higher prevalence of positivity for *T. cruzi* infection (PR = 4.38, p = 0.004) than individuals who did not report the presence of the vectors inside their houses. This result further underscores the centrality of domestic vector infestation in maintaining transmission cycles and corroborates with previous research identifying in-home triatomine presence as a critical determinant of infection risk [10,11]. The absence of significant associations for other variables, such as general knowledge of the vector or the presence of a chicken coop, reinforces the primacy of direct vector contact in transmission dynamics.

Methodologically, the use of IBMP chimeric antigens for serological screening, complemented by commercial assays for case validation, resulted in high diagnostic accuracy. The application of latent class analysis to classify seropositive cases minimized misclassification bias and enhanced the reliability of our findings. These methodological strengths demonstrate the utility of integrating novel diagnostic tools with community-based health initiatives to effectively address CD in endemic regions.

Although most participants were aware of their clinical condition, it is noteworthy that 23% of individuals identified as *T. cruzi*-positive were unaware of their infection status. This finding highlights the insidious nature of chronic CD, which can remain asymptomatic or manifest with nonspecific symptoms for extended periods, thereby complicating timely diagnosis. Limited access to healthcare services, suboptimal screening practices, and low disease awareness within at-risk communities may contribute to this diagnostic gap. The fact that nearly a quarter of seropositive individuals were previously undiagnosed underscores the need for enhanced community-based screening programs, particularly in endemic areas. By integrating serological testing with cardiac screening initiatives, as demonstrated in our study, early detection and

intervention become feasible, potentially curtailing disease progression and reducing long-term morbidity. This observation aligns with earlier research that has documented substantial rates of undiagnosed infections in similar settings, thereby reinforcing the imperative to improve public health strategies aimed at the early identification and management of CD.

However, the cross-sectional design of this study limits causal inferences, and the relatively small number of seropositive cases may affect the generalizability of our findings. Additionally, the recruitment of participants through a cardiac screening initiative introduces a potential selection bias, as individuals with underlying cardiovascular abnormalities may not be representative of the broader population at risk for *T. cruzi* infection. This could lead to an overestimation of CD seropositivity rate in this specific subgroup while underrepresenting asymptomatic or early-stage infections in the general community. To mitigate this limitation, we employed an AI-driven algorithm to randomly select controls, minimizing bias in serological testing and improving the representativeness of the study sample. Future longitudinal studies with larger, community-based samples, such as those planned within the Oxente Chagas Project [15], are needed to explore the temporal dynamics of infection risk and validate these findings in broader populations.

In conclusion, our study provides critical insights into the epidemiology of *T. cruzi* infection in Feira de Santana, Bahia, emphasizing the central role of vector exposure in CD transmission. The integration of seroepidemiological surveys with cardiac screening initiatives demonstrates a valuable model for identifying at-risk populations and enhancing early detection of CD. Continued investment in vector control, diagnostic innovation, and public health education is essential to reduce the burden of CD in endemic regions. These findings underscore the importance of community-based health initiatives

in advancing CD control and improving outcomes in underserved and vulnerable populations.

Acknowledgments. We are grateful to the Holy House of Mercy team in Feira da Santana for their invaluable assistance in performing this study.

References

1. Laporta GZ, Lima MM, Maia da Costa V, de Lima Neto MM, Palmeira SL, Rodovalho SR, et al. Estimativa de prevalência de doença de Chagas crônica nos municípios brasileiros. Revista Panamericana de Salud Publica. 2024;48: e28. doi:10.26633/RPSP.2024.28
2. Santos EF, Silva ÂAO, Leony LM, Freitas NEM, Daltro RT, Regis-Silva CG, et al. Acute Chagas disease in Brazil from 2001 to 2018: A nationwide spatiotemporal analysis. PLoS Negl Trop Dis. 2020;14: e0008445. doi:10.1371/journal.pntd.0008445
3. Chaves GC, Abi-Saab Arrieche M, Rode J, Mechali D, Reis PO, Alves RV, et al. Estimating demand for anti-Chagas drugs: a contribution for access in Latin America. Pan American Journal of Public Health. 2017;41: e45. doi:10.26633/RPSP.2017.45
4. Carvalho CMM de, Ribeiro-Jr G, Gurgel-Gonçalves R, Andrade LS, Moraes CA, Figueiredo MAA. Spatio-temporal trends in mortality due to Chagas disease in the State of Bahia, Brazil, from 2008 to 2018. Rev Soc Bras Med Trop. 2024;57: e004172024. doi:10.1590/0037-8682-0058-2024
5. Diamantino AC, Nascimento BR, Nunes MCP, Sable CA, Oliveira KKB, Rabelo LC, et al. Impact of incorporating echocardiographic screening into a clinical prediction model to optimise utilisation of echocardiography in primary care. Int J Clin Pract. 2021;75: e13686. doi:10.1111/ijcp.13686
6. Santos FLN, Celedon PAF, Zanchin NIT, Brasil TAC, Foti L, Souza WV, et al. Performance assessment of four chimeric Trypanosoma cruzi antigens based on antigen-antibody detection for diagnosis of chronic Chagas disease. PLoS One. 2016;11: e0161100. doi:10.1371/journal.pone.0161100
7. Santos FLN, Campos ACP, Amorim LDAF, Silva ED, Zanchin NIT, Celedon PAF, et al. Highly accurate chimeric proteins for the serological diagnosis of chronic Chagas disease: A latent class analysis. Am J Trop Med Hyg. 2018;99: 1174–1179. doi:10.4269/ajtmh.17-0727
8. Santos EF, Leony LM, Silva ÂAO, Daltro RT, Freitas NEM, Vasconcelos LCM, et al. Assessment of Liaison XL Murex Chagas diagnostic performance in blood screening for Chagas disease using a reference array of chimeric antigens. Transfusion (Paris). 2021;61: 2701–2709. doi:10.1111/trf.16583
9. Santos EF, Silva ÂAO, Freitas NEM, Almeida MCC, Araújo FLV, Celedon PAF, et al. Performance of chimeric Trypanosoma cruzi antigens in serological screening for

Chagas disease in blood banks. Front Med (Lausanne). 2022;9: 852864. doi:10.3389/fmed.2022.852864

10. World Health Organization. Chagas Disease (American Trypanosomiasis). In: https://www.who.int/health-topics/chagas-disease. 21 Oct 2023.

11. Dias JCP, Ramos AN, Gontijo ED, Luquetti A, Shikanai-Yasuda MA, Coura JR, et al. 2 nd Brazilian Consensus on Chagas Disease, 2015. Rev Soc Bras Med Trop. 2016;49: 3–60. doi:10.1590/0037-8682-0505-2016

12. Pavan TBS, Dias DP, Cangussú MM, Dutra VPP, Sampaio DD, Santos FLN. Seroepidemiology of Chagas disease in at-risk individuals in Caraíbas, a city with high endemicity in Bahia State, Brazil. Front Public Health. 2023;11. doi:10.3389/fpubh.2023.1196403

13. Escolano P, Liporaci N, Manzan C, Barbosa A, Alves V, Teixeira R, et al. Prevalence of Chagas infection in Catolândia-Bahia. Rev Soc Bras Med Trop. 1989;22: 159–60. doi:10.1590/s0037-86821989000300009

14. Aras R, Gomes I, Veiga M, Melo A. Transmissão vetorial da doença de Chagas em Mulungu do Morro, Nordeste do Brasil. Rev Soc Bras Med Trop. 2003;36: 359–63. doi:10.1590/s0037-86822003000300008

15. Santos FLN, Pavan TBS, Valle CS, Sampaio DD, Vasconcelos LCM, Cristóbal MH, et al. The Oxente Chagas Bahia Project: Evaluating the efficacy of a rapid diagnostic test and treatments for Chagas disease. Mem Inst Oswaldo Cruz. 2024;119: e240140.

Figure 1

Click here to access/download;Figure;Fig1.tif

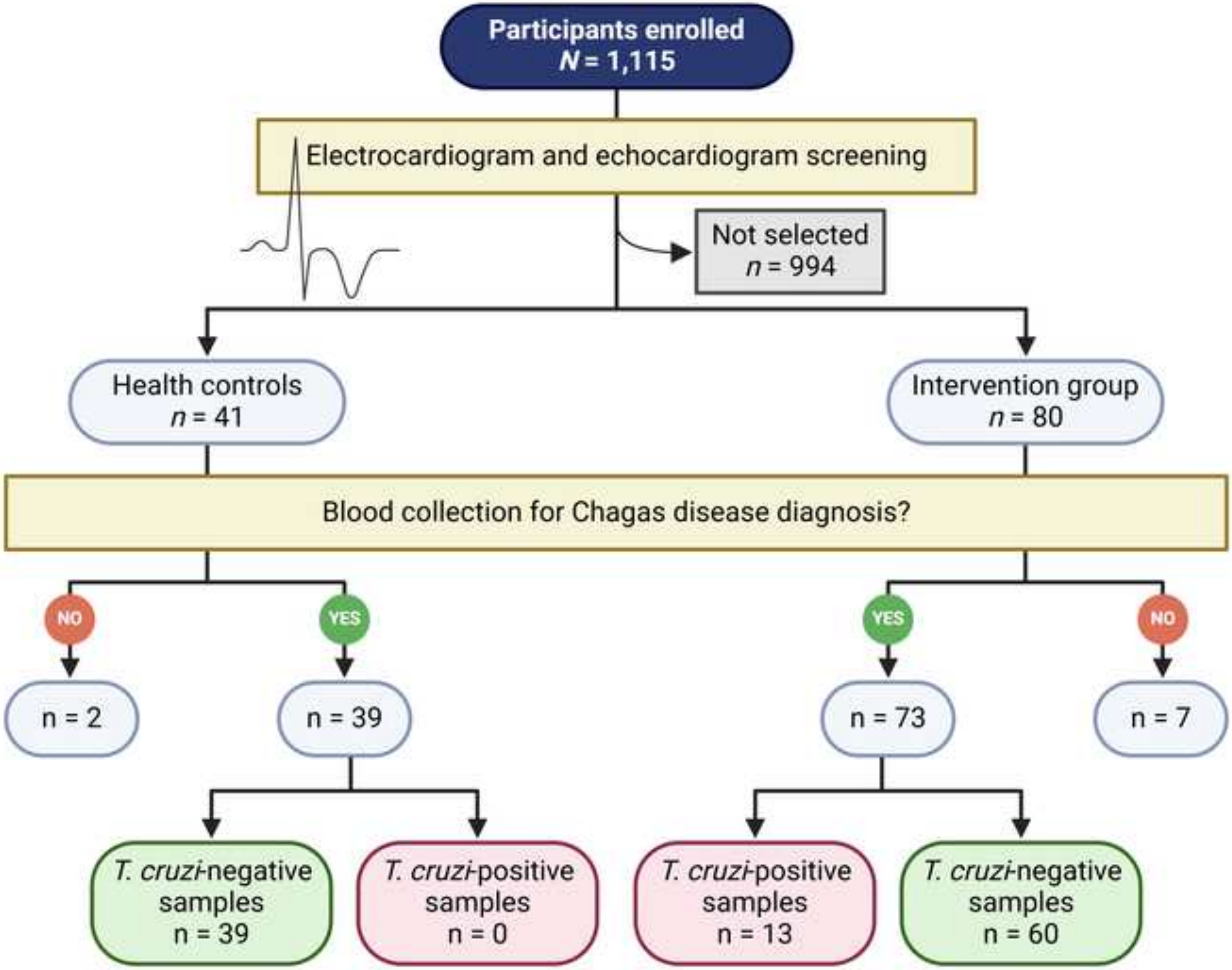

Figure 2

Click here to access/download;Figure;Fig2.tif

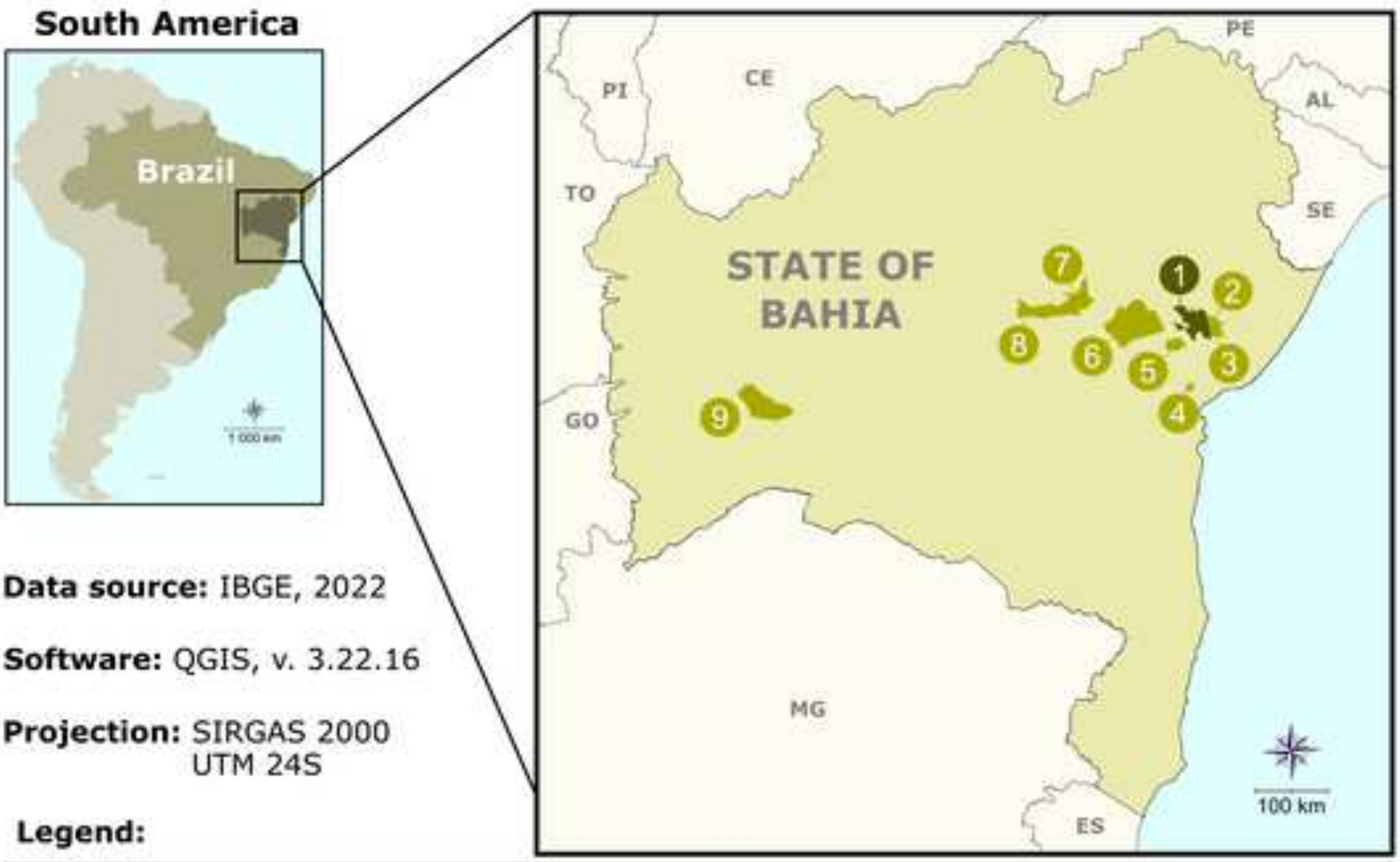

Data source: IBGE, 2022

Software: QGIS, v. 3.22.16

Projection: SIRGAS 2000 UTM 24S

Legend:

	Birthplace	Participant number	Sex	Age (in years)	Occupation	Previous CD awareness
1	Feira de Santana	1	Male	9	Student	Yes
		2	Female	13	Student	Yes
		3	Female	73	Retired	Yes
		4	Female	64	Retired	No
2	Coração de Maria	5	Female	61	Washerwoman	Yes
3	Conceição do Jacuípe	6	Female	77	Retired	No
4	Muniz Ferreira	7	Female	73	Retired	Yes
5	Santo Estevão	8	Male	40	Unemployed	Yes
		9	Female	38	Housewife	Yes
6	Ipirá	10	Female	65	Retired	Yes
7	Mundo Novo	11	Female	59	Retired	Yes
8	Utinga	12	Female	60	Housewife	No
9	Santa Maria da Vitória	13	Female	46	Welfare recipient	Yes
