# Supplementary material for: Seroepidemiological survey and seropositivity rate for Trypanosoma cruzi infection in a community-based cardiac screening initiative in Feira de Santana, Bahia, Brazil
Source: PLoS Negl Trop Dis. 2026 Jan 2;20(1):e0013892. doi: 10.1371/journal.pntd.0013892 (PMC12782403; doi:10.1371/journal.pntd.0013892)
Supplement: Attachment — Submitted filename: Response to Reviewers.pdf [file pntd.0013892.s006.pdf]

Ministério da Saúde
FIOCRUZ
Fundação Oswaldo Cruz
Instituto Gonçalo Moniz

Salvador, Brazil - December 2025

Dear Editors,
PLos Neglected Tropical Diseases

We would like to thank you and the reviewers for the careful, thorough, and constructive evaluation of our manuscript (Manuscript ID: PNTD-D-25-00569R1). We greatly appreciate the time and expertise dedicated to the review process, which has substantially strengthened the scientific rigor, clarity, and transparency of our study.

In response to the Editor's recommendation and Reviewer #3's key concern regarding the absence of a second serological test to confirm infection, we performed an additional independent serological assay. Specifically, all 112 serum samples included in the study were re-evaluated using an indirect hemagglutination assay (IHA; Imuno-HAI Chagas, WAMA Diagnóstica, Brazil). Importantly, the IHA results were fully concordant with the infection status previously established by latent class analysis (LCA), providing independent confirmation of the serological classification and reinforcing the robustness of the diagnostic framework underpinning the study. The Methods, Results, Abstract, and Discussion sections were revised accordingly to explicitly incorporate and describe this additional confirmatory step.

We also carefully addressed the concern regarding the analysis of triatomine exposure data. As acknowledged in the revised manuscript, questionnaire data on vector exposure were not systematically collected across the entire screened population, which limits causal inference in the risk factor analysis. This limitation is now more clearly contextualized in relation to the primary objective of the study, which was to evaluate the feasibility of integrating seroepidemiological screening into a large-scale community-based cardiac screening initiative. We further emphasize that this methodological approach will be formally validated in future population-based and longitudinal studies conducted in different epidemiological contexts.

In addition, following Reviewer #2's recommendation, the entire manuscript—including the reference list—was carefully revised to ensure conceptual accuracy, particularly by consistently referring to transmission of the etiological agent Trypanosoma cruzi rather than transmission of Chagas disease itself. To further improve clarity and readability, the manuscript was reviewed by a native English speaker, and all language-related modifications are highlighted in green in the file Manuscript_with_tracks.

We believe that these substantial revisions directly address the reviewers' and Editor's concerns and significantly enhance the methodological rigor and clarity of the manuscript. We respectfully submit this revised version for your reconsideration and

sincerely hope it will now be suitable for publication in PLOS Neglected Tropical Diseases.

Thank you again for your consideration.

Sincerely,

Fred Luciano Neves Santos
Corresponding author
fred.santos@fiocruz.br

Editors' Recommendations

Additional Editor Comments. The revised manuscript represents a significant improvement, but we need an explicit consideration regarding the concern about "the absence of a second serological test to confirm infection" raised by Reviewer #3. Could the lack of such a confirmatory test have biased the results of the study?

Reply: We thank the Editor and Reviewer #3 for raising this important point. We agree that the absence of an explicit confirmatory serological test could raise concerns about potential bias in infection classification. To address this issue, we performed an additional indirect hemagglutination assay (IHA; Imuno-HAI Chagas, WAMA Diagnóstica, Brazil) to further characterize all 112 serum samples included in the study. The IHA results were fully concordant with the infection status previously determined by latent class analysis (LCA), providing additional confirmation of the serological classification. The manuscript has been revised accordingly, and the relevant methodological details and results have been incorporated throughout the text.

Journal Requirements. If the reviewer comments include a recommendation to cite specific previously published works, please review and evaluate these publications to determine whether they are relevant and should be cited. There is no requirement to cite these works unless the editor has indicated otherwise.

Reply: We thank the editors for their careful evaluation of the manuscript. At this stage of the review process, the reviewers did not request the inclusion or evaluation of any additional previously published references. Therefore, no new citations were added to the manuscript.

Journal Requirements. Kindly revise your competing statement in the online submission form to align with the journal's style guidelines: 'The authors declare that there are no competing interests.'

Reply: We have revised the competing interests statement in the online submission form to align with the journal's style guidelines. The statement now reads: "The authors declare that there are no competing interests."

Figure resubmission. While revising your submission, we strongly recommend that you use PLOS's NAAS tool (https://ngplosjournals.pagemajik.ai/artanalysis) to test your figure files. NAAS can convert your figure files to the TIFF file type and meet basic requirements (such as print size, resolution), or provide you with a report on issues that do not meet our requirements and that NAAS cannot fix.

Reply: We thank the editors for the recommendation regarding the use of the PLOS NAAS tool. No figures were resubmitted in this revision, and therefore no additional figure files required validation or conversion at this stage.

Reviewer #1

Methods. Reviewer #1: The manuscript was greatly improved. The objectives are clear and the design is appropriate to address the objectives. The manuscript presents sufficient sample size to answer the objectives. All concerns about ethical or regulatory requirements were clarified.

Reply: We thank Reviewer #1 for the positive and constructive feedback.

Results. Reviewer #1: The results are clear and match the analysis plan and are presented in full. Figs and tables are sufficient to illustrate the data obtained.

Reply: We thank Reviewer #1 for the positive and constructive feedback.

Conclusions. Reviewer #1: The conclusion is now addressing all relevant results obtained in the revised study encompassing an important topic on public heath once it updates the seroepidemiological and seropositivity data in Feira de Santana, BA, Brazil. The obtained data was clearly discussed and contextualized in terms of advances and understanding Chagas disease in the studied area.

Reply: We thank Reviewer #1 for the positive and constructive feedback.

Summary and General Comments. Reviewer #1: All issues were already addressed.

Reply: We thank Reviewer #1 for the positive and constructive feedback.

Reviewer #2

Methods. Reviewer #2: The objectives are clear and consistent with the cross-sectional study design. In this study, the sample population corresponds to the tested hypothesis, and the statistical analysis supports the conclusions. There are no ethical concerns.

Reply: We thank Reviewer #2 for the positive and constructive feedback.

Results. Reviewer #2: The data analysis corresponds to the study plan, which involved investigating the prevalence of *Trypanosoma cruzi* infection and associated risk factors in an area endemic for Chagas disease in the state of Bahia, Brazil using a cardiac screening program. The seropositivity rate in this area is 8.5%. The data are clear and complete; figures and tables are self-explanatory and of a high standard.

Reply: We thank Reviewer #2 for the positive and constructive feedback.

Conclusions. Reviewer #2: The conclusions are appropriate to the data emphasizing the study limitations to study cross-sectional design. The study emphasizes the significance of vector exposure in the transmission of Chagas disease, underscoring the necessity of targeted

interventions. Further longitudinal studies involving larger community-based samples are required to investigate the temporal dynamics of infection risk by the parasite, and to validate these findings within broader populations.

Reply: We thank Reviewer #2 for the positive and constructive feedback.

Summary and General Comments. Reviewer #2: I suggest that the authors review the entire manuscript, including the references. I recommend stating that transmission is of the etiological agent, *T. cruzi*, rather than of the disease itself, since most individuals with positive serology remain asymptomatic.

Reply: We thank Reviewer for this important conceptual clarification. We agree that transmission refers to the etiological agent *Trypanosoma cruzi* rather than to Chagas disease itself, as most seropositive individuals remain asymptomatic. Accordingly, the manuscript was carefully reviewed in its entirety, including the reference list, and the wording was revised throughout the text to consistently refer to transmission of *T. cruzi* where appropriate. In addition, the manuscript was reviewed by a native English speaker to improve clarity, fluency, and overall readability. All modifications resulting from this language revision are highlighted in green in the file Manuscript_with_tracks.

Reviewer #3

Methods. Reviewer #3: The main point made by me in the previous version was not answered: the absence of a second serological test to confirm infection. This absence impacts on the entire article because it lacks the necessary robustness to define those infected humans. Another unresolved point is associating the questionnaire on triatomine exposure only with serologically positive and not negative tests. The authors agreed and included this as a limitation, but simply including this limitation does not validate the analysis. Because I understand the first aspect impacts the accuracy of the diagnosis on which the entire article is based, I maintain my recommendation to reject the article.

Reply: We thank the Editor and Reviewer #3 for raising this important point. We agree that the absence of an explicit confirmatory serological test could raise concerns regarding potential bias in infection classification. To address this issue, we performed an additional indirect hemagglutination assay (IHA; Imuno-HAI Chagas, WAMA Diagnóstica, Brazil) to further characterize all 112 serum samples included in the study. The IHA results were fully concordant with the infection status previously determined by latent class analysis (LCA), providing additional confirmation of the serological classification. The manuscript has been revised accordingly, and the relevant methodological details and results have been incorporated throughout the text. With regard to the association between triatomine exposure and serological status, we acknowledge that the questionnaire data on vector exposure were not systematically collected or compared between all seropositive and seronegative individuals in the entire screened population. As discussed in the revised manuscript, this limitation restricts causal inference and the generalizability of the risk factor analysis. However, the primary

objective of the present study was to assess the feasibility of integrating seroepidemiological screening into a large-scale cardiac screening initiative and to explore associated epidemiological patterns in this specific context. The methodological approach proposed here will be further evaluated and refined in future validation studies, including broader population-based designs and different epidemiological settings, in order to formally assess risk factors and validate the applicability of this strategy across diverse populations.
