# Supplementary material for: Seroepidemiological survey and seropositivity rate for Trypanosoma cruzi infection in a community-based cardiac screening initiative in Feira de Santana, Bahia, Brazil
Source: PLoS Negl Trop Dis. 2026 Jan 2;20(1):e0013892. doi: 10.1371/journal.pntd.0013892 (PMC12782403; doi:10.1371/journal.pntd.0013892)
Supplement: Attachment — Submitted filename: Response to Reviewers.docx [file pntd.0013892.s005.docx]

Salvador, Brazil - September 2025

**Dear Editors,**

PLos Neglected Tropical Diseases

We would like to express our sincere gratitude to you and to the reviewers for the thoughtful and constructive recommendations provided. The feedback has been invaluable in improving the quality and clarity of our manuscript. We believe that the revised version now presents a more accurate and comprehensive account of our research.

We are confident that this version aligns with the high standards of Plos Neglected Tropical Diseases, and we kindly request your consideration for publication. For ease of reference, we have provided detailed responses to the reviewers’ comments below (Manuscript ID: PNTD-D-25-00569).

Additionally, we respectfully request authorization to adjust the order of authors. Dr. Antônio Luiz Pinho Ribeiro, who was responsible for the clinical analysis of the participants, should be placed in a prominent position at the end of the authorship list (before me) to reflect specific contribution appropriately.

Thank you very much for your attention and consideration.

Sincerely,

Fred Luciano Neves Santos

Corresponding author

fred.santos@fiocruz.br

**Editors' Recommendations**

**Journal Requirements.** We do not publish any copyright or trademark symbols that usually accompany proprietary names, eg ©, ®, or TM (e.g. next to drug or reagent names). Therefore, please remove all instances of trademark/copyright symbols throughout the text, including: ® on pages: 7, 9, and 10.

**Reply:** We sincerely thank the Editors for this guidance. In accordance with the journal’s formatting requirements, we have carefully removed all instances of trademark and copyright symbols throughout the manuscript, including the ® symbols identified on pages 7, 9, and 10.

*Before*: “…commercial assays, including Gold ELISA Chagas® (REM Indústria e Comércio Ltda, Brazil) and Bioelisa Chagas Recombinante® (Bioclin Quibasa Química Básica, Brazil).”

*After* (line 183-184): “…commercial assays, including Gold ELISA Chagas (REM Indústria e 76Comércio Ltda, Brazil) and Bioelisa Chagas Recombinante (Bioclin Quibasa Química Básica, Brazil).”

*Before*: “…with Gold ELISA Chagas® and Bioelisa Chagas Recombinante® detecting seropositivity in 100% (13/13) of cases.”

*After* (line 243-244): “…with Gold ELISA Chagas and Bioelisa Chagas Recombinante detecting seropositivity in 100% (13/13) of cases.”

*Before*: “^1^GEC (Gold ELISA Chagas®); ^2^BIO (Bioelisa Chagas Recombinante®); LCA...”

*After* (line 252): “^1^GEC (Gold ELISA Chagas); ^2^BIO (Bioelisa Chagas Recombinante); LCA…”

**Figure resubmission.** While revising your submission, please upload your figure files to the Preflight Analysis and Conversion Engine (PACE) digital diagnostic tool, https://pacev2.apexcovantage.com/. PACE helps ensure that figures meet PLOS requirements. To use PACE, you must first register as a user. Registration is free. Then, login and navigate to the UPLOAD tab, where you will find detailed instructions on how to use the tool. If you encounter any issues or have any questions when using PACE, please email PLOS at figures@plos.org. Please note that Supporting Information files do not need this step. If there are other versions of figure files still present in your submission file inventory at resubmission, please replace them with the PACE-processed versions.

**Reply:** We confirm that all figure files have been submitted to the PACE (Preflight Analysis and Conversion Engine) system as instructed. The figures were successfully processed and verified as compliant with the journal’s formatting requirements. The PACE-processed versions have been uploaded to replace the previous files in the submission inventory.

**Reviewer #1**

This is a very relevant manuscript, which updates and integrates a seroepidemiological survey with large-scale echocardiographic screening to estimate the prevalence of *T. cruzi* infection and identify associated risk factors in Feira de Santana, Bahia, Brazil. The manuscript is well written, presented, the methodology is in accordance with the objectives, and results. The main concern is the short introduction. A few other issues were also detected; therefore, changes are recommended before the final acceptance of the manuscript in the PNTD.

**Abstract.** Please provide brief information on the place where the study was carried out.

**Reply:** We thank the reviewer for this valuable suggestion. In response, we have revised the Abstract to include the study location, thereby providing essential geographic context. The revised text now specifies that the study was conducted in Feira de Santana, Bahia, Brazil, a highly endemic region for Chagas disease.

*Before (original Abstract opening)*: “Chagas disease (CD), caused by *Trypanosoma cruzi*, is a significant public health issue in Latin America, particularly in endemic regions. This study integrates a seroepidemiological survey with large-scale echocardiographic screening to estimate the prevalence of *T. cruzi* infection and identify associated risk factors.”

*After* (revised Abstract opening; line 43-44): “Chagas disease (CD), caused by *Trypanosoma cruzi*, is a significant public health issue in Latin America, particularly in endemic regions. This study integrates a seroepidemiological survey with large-scale echocardiographic screening conducted in Feira de Santana, a highly endemic city in Bahia, Brazil, to estimate the prevalence of *T. cruzi* infection and identify associated risk factors.”

**Specific comments.** Please provide brief background on Bahia State related to epidemiological surveys on Chagas disease as well as for Feira de Santana. The authors cite reference n. 4 however, we recommend a few words on the CD in Bahia and specially in Feira de Santana Municipality.

**Reply:** We thank the reviewer for this thoughtful and constructive suggestion. In response, we have revised the Introduction to provide additional background on the epidemiological scenario of Chagas disease in Bahia State and in Feira de Santana specifically. These additions help situate our study within the broader regional context and emphasize the local relevance of our findings.

*Before (original Introduction excerpt)*: “Feira de Santana, Bahia, is a highly CD endemic region in Brazil with a substantial burden, where Chagas-related mortality significantly impacts local health [4]. Socioeconomic disparities and inadequate healthcare infrastructure exacerbate diagnostic and treatment delays.”

*After* (revised Introduction excerpt; line 81-92): “Bahia State is recognized as one of the Brazilian states with persistent endemicity for Chagas disease, where both vectorial and oral transmission have been historically documented, and recent surveys continue to report significant seroprevalence rates in rural communities [4–6]. The state also harbors the richest biodiversity of triatomine fauna in Brazil, being one of the most infested regions in the country. In Bahia, new species have been described or revalidated, and several triatomine species have shown adaptation to human dwellings, further enhancing the risk of vectorial transmission [7–10]. Within Bahia, Feira de Santana is one of the most populous municipalities and plays a central role as a regional hub. The municipality has reported substantial morbidity and mortality related to CD, reflecting the challenges of disease control and patient management in the state [11]. Socioeconomic disparities and inadequate healthcare infrastructure further exacerbate diagnostic and treatment delays in this region.”

*References*:

4. Pavan TBS, et al. Seroepidemiology of Chagas disease in at-risk individuals in Caraíbas, a city with high endemicity in Bahia State, Brazil. Front Public Health. 2023;11. doi:10.3389/fpubh.2023.1196403

5. Escolano P, et al. Prevalence of Chagas infection in Catolândia-Bahia. Rev Soc Bras Med Trop. 1989;22: 159–60. doi:10.1590/s0037-86821989000300009

6. Aras R, et al. Transmissão vetorial da doença de Chagas em Mulungu do Morro, Nordeste do Brasil. Rev Soc Bras Med Trop. 2003;36: 359–63. doi:10.1590/s0037-86822003000300008

7. Ribeiro-Jr G, et al. Triatomine fauna in the state of Bahia, Brazil: What changed after 40 years of the vector-control program? Rev Soc Bras Med Trop. 2022;55: e07322021. doi:10.1590/0037-8682-0732-2021

8. Costa J, et al. Do the new triatomine species pose new challenges or strategies for monitoring chagas disease? An overview from 1979-2021. Mem Inst Oswaldo Cruz. 2021;116: e210015. doi:10.1590/0074-02760210015

9. Gurgel-Gonçalves R, et al. Geographic distribution of chagas disease vectors in Brazil based on ecological niche modeling. J Trop Med. 2012;2012. doi:10.1155/2012/705326

10. Costa J, et al. The epidemiologic importance of Triatoma brasiliensis as a Chagas disease vector in Brazil: A revision of domiciliary captures during 1993-1999. Mem Inst Oswaldo Cruz. 2003;98: 443–449. doi:10.1590/S0074-02762003000400002

11. Carvalho CMM, et al. Spatio-temporal trends in mortality due to Chagas disease in the State of Bahia, Brazil, from 2008 to 2018. Rev Soc Bras Med Trop. 2024;57: e004172024. doi:10.1590/0037-8682-0058-2024

**Specific comments.** Also, it is recommended that there are some words on the triatomine fauna in Bahia State, which presents the richest biodiversity of CD vector species, being one of the most infested states in Brazil. In addition to that, new species have been described, revalidated in that state and also new triatomine species have been adapted to the human dwellings enhancing the risk of transmission of Chagas disease.

**Reply:** We thank the reviewer for this valuable observation. In response, we have expanded the Introduction to highlight that Bahia State harbors the richest biodiversity of triatomine fauna in Brazil and is among the most heavily infested regions in the country. We also incorporated information on the description and revalidation of new species, as well as the adaptation of several triatomine species to human dwellings, which increases the risk of vectorial transmission. These additions strengthen the ecological and epidemiological context of our study by situating it within a high-risk region for Chagas disease transmission.

*After* (revised Introduction excerpt; line 84-88): “The state also harbors the richest biodiversity of triatomine fauna in Brazil, being one of the most infested regions in the country. In Bahia, new species have been described or revalidated, and several triatomine species have shown adaptation to human dwellings, further enhancing the risk of vectorial transmission [7–10]..”

*References:*

7. Ribeiro-Jr G, et al. Triatomine fauna in the state of Bahia, Brazil: What changed after 40 years of the vector-control program? Rev Soc Bras Med Trop. 2022;55: e07322021. doi:10.1590/0037-8682-0732-2021

8. Costa J, et al. Do the new triatomine species pose new challenges or strategies for monitoring chagas disease? An overview from 1979-2021. Mem Inst Oswaldo Cruz. 2021;116: e210015. doi:10.1590/0074-02760210015

9. Gurgel-Gonçalves R, et al. Geographic distribution of chagas disease vectors in Brazil based on ecological niche modeling. J Trop Med. 2012;2012. doi:10.1155/2012/705326

10. Costa J, et al. The epidemiologic importance of Triatoma brasiliensis as a Chagas disease vector in Brazil: A revision of domiciliary captures during 1993-1999. Mem Inst Oswaldo Cruz. 2003;98: 443–449. doi:10.1590/S0074-02762003000400002

**Specific comments.** The authors mention the vectors as a risk factor for CD therefore, it is recommended including some references mentioning the vector species in Bahia.

Costa J, Almeida CE, Dotson EM, Lins A, Vinhaes M, Silveira AC, et al. The epidemiologic importance of Triatoma brasiliensis as a Chagas disease vector in Brazil: a revision of domiciliary captures during 1993-1999. Mem Inst Oswaldo Cruz. 2003; 98(4): 443-9.

Gurgel-Gonçalves R, Galvão C, Costa J, Peterson AT. Geographic distribution of Chagas disease vectors in Brazil based on ecological niche modeling. J Trop Med. 2012; 705326.

Costa, J.; Dale, C.; Galvão, C.; Almeida, C.E.; Dujardin, J.P. Do the new triatomine species pose new challenges or strategies for monitoring Chagas disease? An overview from 1979–2021. Mem. Inst. Oswaldo Cruz 2021, 116, e210015.

**Reply:** We thank the reviewer for this helpful recommendation. In response, we have revised the Introduction to incorporate additional references that address the epidemiological importance and geographic distribution of triatomine species in Bahia. These references reinforce the contextual background and support our emphasis on vector exposure as a key risk factor for Chagas disease transmission in the region.

*After* (revised Introduction excerpt; line 84-88): “The state also harbors the richest biodiversity of triatomine fauna in Brazil, being one of the most infested regions in the country. In Bahia, new species have been described or revalidated, and several triatomine species have shown adaptation to human dwellings, further enhancing the risk of vectorial transmission [7–10]..”

*References:*

7. Ribeiro-Jr G, et al. Triatomine fauna in the state of Bahia, Brazil: What changed after 40 years of the vector-control program? Rev Soc Bras Med Trop. 2022;55: e07322021. doi:10.1590/0037-8682-0732-2021

8. Costa J, et al. Do the new triatomine species pose new challenges or strategies for monitoring chagas disease? An overview from 1979-2021. Mem Inst Oswaldo Cruz. 2021;116: e210015. doi:10.1590/0074-02760210015

9. Gurgel-Gonçalves R, et al. Geographic distribution of chagas disease vectors in Brazil based on ecological niche modeling. J Trop Med. 2012;2012. doi:10.1155/2012/705326

10. Costa J, et al. The epidemiologic importance of Triatoma brasiliensis as a Chagas disease vector in Brazil: A revision of domiciliary captures during 1993-1999. Mem Inst Oswaldo Cruz. 2003;98: 443–449. doi:10.1590/S0074-02762003000400002

**Material and Methods.** Please provide relevant information about Feira de Santana, physio geographic information based for instance in IBGE data and more specifically about the place the analyzed people reside, age, sex, socio economic status etc.

**Reply:** We thank the reviewer for this valuable suggestion. In response, we have revised the Materials and Methods section to include physiogeographic information on Feira de Santana, based on IBGE data, as well as more detailed sociodemographic characteristics of the study population. These additions provide important context regarding the setting and the social conditions in which the study was conducted.

*Before (original Methods – Study population excerpt)*: “This cross-sectional study included 1,115 adults who were referred for a large-scale screening, which comprised a structured questionnaire, electrocardiogram (ECG), and echocardiography.”

*After* (revised Methods – Study population excerpt; line 109-127): “This cross-sectional study was conducted in Feira de Santana, Bahia, Brazil. Feira de Santana is the second largest city in Bahia, located in the semi-arid region of the Northeast (11° 11′ S, 38° 58′ W) at an altitude of 234 meters, with a tropical savanna climate characterized by a prolonged dry season. According to the Brazilian Institute of Geography and Statistics (IBGE), the municipality has a population of approximately 616,272 inhabitants, making it an important commercial and transport hub in the state [13]. The urban area is characterized by high population density and marked social inequalities, with peripheral neighborhoods presenting limited access to health services, sanitation, and housing infrastructure.

A total of 1,115 adults were recruited for participation in a large-scale screening initiative. All participants completed a structured questionnaire that captured sociodemographic information (age, sex, education level, employment status, household income), environmental and housing conditions (type of dwelling materials, presence of domestic animals, chicken coops, and history of triatomine sightings), family history of Chagas disease, migration background, and previous medical diagnoses. In addition, cardiovascular symptoms were assessed by asking about exercise tolerance, chest pain, syncope, palpitations, and other relevant symptoms. The questionnaire was administered by trained healthcare professionals or supervised medical students at the time of the echocardiographic examination.

*Reference:*

Instituto Brasileiro de Geografia e Estatística (IBGE). Feira de Santana. In: IBGE Cidades [Internet]. 2023 [cited 25 Sep 2025]. Available: https://cidades.ibge.gov.br/brasil/ba/feira-de-santana/panorama

**Discussion.** From lines 312 – 315 please, include a reference in the text.

**Reply:** We thank the reviewer for this valuable suggestion. In the revised version of the manuscript, we have added relevant references to strengthen the statement regarding the prevalence of undiagnosed infections in similar endemic settings.

*Revised Discussion excerpt (lines 356-359):* This observation aligns with earlier research that has documented substantial rates of undiagnosed infections in similar settings, thereby reinforcing the imperative to improve public health strategies aimed at the early identification and management of CD [23–25].

*References added:*

23. Fidalgo ASOBV, et al. Seroprevalence and risk factors of Chagas disease in a rural population of the Quixeré municipality, Ceará, Brazil. Rev Soc Bras Med Trop. 2021;54: 1–6. doi:10.1590/0037-8682-0247-2020

24. Freitas EC, et al. Analysis of the seroprevalence of and factors associated with Chagas disease in an endemic area in Northeastern Brazil. Rev Soc Bras Med Trop. 2017;50: 44–51. doi:10.1590/0037-8682-0242-2016

25. Aquino Santana M, et al. Seroprevalence of Chagas disease in rural communities at Campinas do Piauí city, Brazil. Tropical Medicine and International Health. 2021;26: 281–289. doi:10.1111/tmi.13516

**Discussion.** From lines 333-335 please, include a reference in the text.

**Reply:** We thank the reviewer for this valuable suggestion. In response, we have included additional references to substantiate our statement regarding the need for sustained investment in vector control, diagnostic innovation, and public health education in endemic areas.

*Revised Discussion excerpt (lines 378-380):* “Continued investment in vector control, diagnostic innovation, and public health education is essential to reduce the burden of CD in endemic regions [22,27].”

*References added:*

22. Dias JCP, et al. 2 nd Brazilian Consensus on Chagas Disease, 2015. Rev Soc Bras Med Trop. 2016;49: 3–60. doi:10.1590/0037-8682-0505-2016

27. Santos FLN, et al. Chagas disease in Brazil: New challenges and perspectives for old problems. Mem Inst Oswaldo Cruz. 2025;120: e240279. doi:10.1590/0074-02760240279

**Figures and Legends.** Legends should be self-explanatory, please revise the legend of fig 1 providing information about the locality, the disease and any other relevant additional information.

**Reply:** We thank the reviewer for this constructive comment. In response, we have revised the legend of Fig 1 to provide clearer and more self-explanatory information, including the study locality (Feira de Santana, Bahia, Brazil), the disease under investigation (Chagas disease), and the type of analysis performed.

*Before (original legend of Fig 1)*: “Fig 1. Study design following the Standards for Reporting of Diagnostic Accuracy Studies (STARD) guidelines.”

*After (revised legend of Fig 1; line 231-236)*: “Fig 1. Study design of a seroepidemiological survey for Chagas disease in Feira de Santana, Bahia, Brazil. The figure summarizes participant selection within the Every Heartbeat Matters cardiac screening initiative, with ECG and echocardiographic evaluation, risk stratification, and subsequent serological testing using IBMP chimeric antigen–based ELISA and commercial assays. The design follows the Standards for Reporting of Diagnostic Accuracy Studies (STARD) guidelines.”

**Figures and Legends.** The same is applied for Table 1, fig 2 and Table 2.

**Reply:** We appreciate this important observation. In response, we have revised the legends of Table 1, Fig 2, and Table 2 to ensure that they are self-explanatory and provide sufficient contextual information regarding the study locality and disease focus.

*Table 1 – Before*: “Table 1. Reactivity patterns and classification of *Trypanosoma cruzi* seropositivity.”

*Table 1 – After (revised legend; line 248-249)*: “Table 1. Reactivity patterns of serum samples from participants screened for Chagas disease in Feira de Santana, Bahia, Brazil.”

*Fig 2 – Before*: “Fig 2. Geographic origins of *Trypanosoma cruzi*-positive individuals in Bahia, Brazil. Public domain digital maps were obtained from the Brazilian Institute of Geography and Statistics (IBGE) cartographic database in shapefile format (.shp), which was subsequently reformatted and analyzed using QGIS version 3.10 (Geographic Information System, Open-Source Geospatial Foundation Project. http://qgis.osgeo.org).”

*Fig 2 – After (revised legend; line 267-272)*: “Fig 2. Geographic origins of individuals seropositive for Trypanosoma cruzi in Feira de Santana, Bahia, Brazil. The map shows the municipalities of birth of participants who tested positive for Chagas disease during the Every Heartbeat Matters screening initiative. Public domain digital maps were obtained from the Brazilian Institute of Geography and Statistics (IBGE) and analyzed using QGIS version 3.10 (Geographic Information System, Open-Source Geospatial Foundation Project. http://qgis.osgeo.org)”.

*Table 2 – Before*: “Table 2. Univariate Analysis of Factors Associated with *Trypanosoma cruzi* Seropositivity.”

*Table 2 – After (revised legend; line 292-293)*: “Table 2. Univariate analysis of epidemiological factors associated with Trypanosoma cruzi seropositivity in participants from Feira de Santana, Bahia, Brazil.”

**Reviewer #2**

This study demonstrates how we can improve diagnostic strategies for *T. cruzi* infection using large-scale digital health AI tools aimed at overcoming barriers to identifying seropositive patients with excellent results. The significant association between household observation and the presence of triatomine highlights the ongoing exposure to the vector and the occurrence of parasite transmission in endemic areas. Given the possibility of increased risk of infection, it is important to emphasize the importance of early diagnosis using innovative methods, prompt etiologic treatment of cases, and well-designed clinical follow-up.

**Editorial and Data Presentation Modifications**. Scientific names should be written in italic letters.

**Reply:** We thank the reviewer and the editorial team for this important observation. In accordance with scientific nomenclature standards, we have carefully revised the entire manuscript to ensure that all scientific names, including *Trypanosoma cruzi* and other relevant taxa, are consistently written in italic formatting.

**Reviewing the list of references.**

**Reply:** We thank the reviewer for this observation. The entire reference list has been carefully reviewed, with all entries verified for accuracy and consistency, and standardized in accordance with the journal’s formatting guidelines.

**Corrections are highlighted in yellow in the text. See attached manuscript.** Please fix LCA to the same line.

**Reply:** We thank the reviewer for the careful revision. All highlighted corrections have been incorporated into the revised manuscript. Additionally, the formatting of “LCA” has been corrected to ensure it appears on the same line for consistency and readability.

**Corrections are highlighted in yellow in the text. See attached manuscript.** Italianize *Trypanosoma cruzi* in reference number 6.

**Reply:** We thank the reviewer for this observation. In the revised manuscript, the genus and species name *Trypanosoma cruzi* in reference number 6 (and others!) has been corrected to italic formatting, in accordance with scientific nomenclature conventions.

**Reviewer #3**

**Methods.** In the absence of a parasitological test, the WHO recommends and is internationally accepted that a definitive serological diagnosis must be based on two distinct serological tests. This is especially important in trypanosomatids, where cross-reactivity with other Trypanosoma species and even Leishmania is reported. The inclusion of a second test should not be presented as “Reference Testing”, but as second confirmatory for the ELISA. Even using four chimeric proteins, it remains the same test (ELISA), and the high chance of cross-reactivity (Leishmaniasis is a reality throughout Brazil) increases the risk of false positives. Morevoer, The echocardiographic test cannot be considered a screening test, given that several other diseases present with cardiac dysfunction, and a high percentage of Chagas seropositive individuals do not develop symptoms.

**Methods.** The “structured questionnaire” (line 100) wasn't presented, and I imagine it's not just the three questions that appear in the following paragraph.

**Reply:** We thank the reviewer for these important observations. Regarding the structured questionnaire: indeed, it contained more items than the three epidemiological questions originally highlighted. In the first version of the manuscript, we reported only those three questions because they were directly integrated into the AI-based risk stratification model. To avoid ambiguity, we have now revised the Methods section to present the broader scope of the questionnaire, which included sociodemographic, socioeconomic, environmental, epidemiological, and clinical information.

*Before (original Methods excerpt, line 100)*: “This cross-sectional study included 1,115 adults who were referred for a large-scale screening, which comprised a structured questionnaire, electrocardiogram (ECG), and echocardiography. An artificial intelligence (AI)-based algorithm was implemented to identify individuals at risk for CD by integrating data from 12-lead ECGs *After* (revised Methods – Study population excerpt; line 109-127): “This cross-sectional study was conducted in Feira de Santana, Bahia, Brazil. Feira de Santana is the second largest city in Bahia, located in the semi-arid region of the Northeast (11° 11′ S, 38° 58′ W) at an altitude of 234 meters, with a tropical savanna climate characterized by a prolonged dry season. According to the Brazilian Institute of Geography and Statistics (IBGE), the municipality has a population of approximately 616,272 inhabitants, making it an important commercial and transport hub in the state [13]. The urban area is characterized by high population density and marked social inequalities, with peripheral neighborhoods presenting limited access to health services, sanitation, and housing infrastructure.

A total of 1,115 adults were recruited for participation in a large-scale screening initiative. All participants completed a structured questionnaire that captured sociodemographic information (age, sex, education level, employment status, household income), environmental and housing conditions (type of dwelling materials, presence of domestic animals, chicken coops, and history of triatomine sightings), family history of Chagas disease, migration background, and previous medical diagnoses. In addition, cardiovascular symptoms were assessed by asking about exercise tolerance, chest pain, syncope, palpitations, and other relevant symptoms. The questionnaire was administered by trained healthcare professionals or supervised medical students at the time of the echocardiographic examination.

*Reference:*

Instituto Brasileiro de Geografia e Estatística (IBGE). Feira de Santana. In: IBGE Cidades [Internet]. 2023 [cited 25 Sep 2025]. Available: https://cidades.ibge.gov.br/brasil/ba/feira-de-santana/panorama

**Methods.** Electrocardiogram (ECG) and echocardiography: Which equipment? How was it performed? By the same professional? Who interpreted the results? The methodological approach is lacking in detail.

**Reply:** We thank the reviewer for this observation. We have revised the Methods section to provide detailed information about the acquisition and interpretation of electrocardiograms (ECG) and echocardiograms. The revised text specifies the equipment used, the professionals involved, and the criteria adopted for classification.

*Revised Methods excerpt (lines 128–137):* “All participants underwent electrocardiogram (ECG) acquired with a PC-based system (TEB, São Paulo, Brazil) and transmitted electronically to the telehealth platform, where cardiologists interpreted them and issued standardized reports. ECG abnormalities were classified as major or minor according to the Minnesota Code criteria [14].

Echocardiograms were performed by a team of eight cardiologists using commercially available systems (Vivid Q, GE Healthcare; Affiniti 70, Philips, USA). Images were stored digitally and interpreted immediately after acquisition; when findings were uncertain, two or more cardiologists conducted a consensus read to establish the final diagnosis. Abnormal echocardiograms were defined by the presence of major abnormalities [15].

*References:*

14. Ribeiro ALP, et al. Electrocardiographic abnormalities in elderly Chagas disease patients: 10-year follow-up of the Bambui Cohort Study of Aging. J Am Heart Assoc. 2014;3: e000632. doi:10.1161/JAHA.113.000632

15. Diamantino AC, et al. Impact of incorporating echocardiographic screening into a clinical prediction model to optimise utilisation of echocardiography in primary care. Int J Clin Pract. 2021;75: e13686. doi:10.1111/ijcp.13686

**Methods.** Artificial intelligence (AI)-based algorithm: How was it done? What was the methodology?

**Reply:** We thank the reviewer for this important observation. To address this, we have revised the Methods section to provide more detail on the artificial intelligence (AI) algorithm used for risk stratification. The revised text specifies the type of model, input data, previous validation, and integration with epidemiological variables.

*Revised Methods excerpt (lines 138-149):* An ECG-based artificial intelligence (AI) algorithm, implemented as a deep neural network and trained on standard 12-lead digital ECG waveforms (time–voltage signals), was used to estimate the risk of positive serology for CD [16]. Based on a combination of epidemiological questions and ECG abnormalities, individuals were categorized into low and high risk for CD. The three selected questions assessed whether participants: (1) had a family member diagnosed with CD, (2) had lived in a region with known triatomine (kissing bug) exposure, and (3) had resided in a wooden house, a known risk factor for vector transmission.

A combination of the answer to these three epidemiological questions and the AI-ECG output was used to generate a risk score, flagging individuals who underwent a serological test for CD. For comparison, controls were randomly selected at a 2:1 ratio (controls to cases) and underwent CD serological testing.

*Reference:*

16. Jidling C, et al. Screening for Chagas disease from the electrocardiogram using a deep neural network. Magalhães L, editor. PLoS Negl Trop Dis. 2023;17: e0011118. doi:10.1371/journal.pntd.0011118

**Methods.** “Clinical data” (line 110): What clinical symptoms formed the basis for the clinical data? Who collected it? What aspects were assessed?

**Reply:** We thank the reviewer for this pertinent question. In the revised manuscript, we have clarified the nature of the “clinical data” collected, specifying the symptoms assessed, the scope of information obtained, and the professionals responsible for collection.

*Revised Methods excerpt (lines 150–155):* “Clinical information was collected using a standardized questionnaire that included sociodemographic characteristics, comorbidities, and history of preexisting heart disease (particularly prior cardiac surgery). Cardiovascular symptoms were assessed by asking participants about exercise tolerance, chest pain, syncope, palpitations, and other relevant manifestations. The questionnaire was administered by trained healthcare professionals or by medical students under direct supervision, at the time of the echocardiogram.”

**Results.** The association with presence of triatomine bugs cannot be done because authors didn’t compare this trait to the negatives. It could just reflect the overall population characteristic.

**Reply:** We thank the reviewer for this important observation. We agree that the association between *T. cruzi* seropositivity and the presence of triatomine bugs must be interpreted with caution, since this trait was not directly compared between positive and negative individuals in the broader screened population. To avoid overstating the finding, we have revised the Results to explicitly acknowledge this limitation and to frame the association more cautiously.

*Before (original Results excerpt)*: “In univariate analysis, household observation of triatomine bugs was significantly associated with *T. cruzi* seropositivity (PR = 4.38, 95% CI: 1.44–13.3, p = 0.004). Other variables, including general knowledge about the vector, the presence of a chicken coop near the house, and a family history of CD, were not significantly associated with seropositivity. This result indicates that individuals who reported seeing these insects indoors had an almost fourfold increase in the likelihood of infection (Table 2).”

*After (revised Results excerpt; line 282-291)*: “In univariate analysis, household observation of triatomine bugs showed a higher prevalence of T. cruzi seropositivity (PR = 4.38, 95% CI: 1.44–13.3, p = 0.004). However, because this trait was not directly compared between positive and negative individuals in the overall screened population, this finding should be interpreted with caution, as it may reflect a general characteristic of the surveyed community rather than a specific risk factor. Other variables, including general knowledge about the vector, the presence of a chicken coop near the house, and a family history of CD, were not significantly associated with seropositivity. This suggests that while reporting triatomine presence indoors may indicate increased risk, the association requires confirmation in broader community-based analyses (Table 2).”

**Results.** Lines 177-178: These results need to be better described. If they are part of another study, you need to reference the other study that contains these data.

**Reply:** We thank the reviewer for this important observation. In response, we have expanded the description of the ECG abnormalities to clarify how they were defined and classified. The classification followed the Minnesota Code Criteria, which distinguishes between major and minor abnormalities. **Major** abnormalities included complete intraventricular conduction blocks (complete right or left bundle-branch block); atrial fibrillation/flutter or sustained supraventricular tachycardia; frequent premature beats (supraventricular or ventricular); major isolated ST-segment or T-wave abnormalities; and high-grade atrioventricular conduction abnormalities (e.g., Mobitz II second-degree or third-degree AV block) or pacemaker rhythm. **Minor** abnormalities included minor isolated Q or QS waves; minor isolated ST-segment or T-wave abnormalities; incomplete bundle-branch block or left anterior fascicular block; minor QT prolongation; short PR interval; left or right axis deviation; and first-degree atrioventricular block. The reference for this classification has been added to the Methods section.

**Results.** The “AI algorithm” (line 179): There is also a lack of information on how it was produced and tested.

**Reply:** We thank the reviewer for this observation. To address it, we have expanded the description of the AI algorithm in the Results and Methods sections to clarify how it was developed, tested, and applied in the present study. An ECG-based artificial intelligence (AI) algorithm was developed as a deep neural network trained on standard 12-lead digital ECG waveforms (time–voltage signals). Prior studies have demonstrated the utility of AI applied to digital ECGs for detecting and predicting cardiovascular disease, including atrial fibrillation, cardiomyopathy, and all-cause mortality. Building on this evidence, our group has previously shown that AI analysis of ECGs can recognize patients with Chagas disease. In the current study, we applied this previously validated ECG-AI alongside epidemiological questions as an initial screening approach to identify patients with Chagas disease (PLoS Negl Trop Dis. 2023;17(9):e0011118. doi:10.1371/journal.pntd.0011118). In the current study, this previously validated ECG-AI was applied in combination with epidemiological questions as an initial screening tool to identify individuals at higher risk for T. cruzi infection. The calibration and large-scale prospective validation of a new ECG-AI specifically designed to detect Chagas disease are ongoing in a separate project and are outside the scope of the present work.

**Results.** “…based on predefined criteria…” (line 181): criteria not presented in the article

**Reply:** We thank the reviewer for this valuable observation. To avoid ambiguity, we have revised the text to clarify how controls were selected. The study used a data-driven approach in which a supervised deep neural network (DNN) learned patterns directly from standard 12-lead digital ECG waveforms (time–voltage signals) to identify individuals at high risk for Chagas disease. Based on the algorithm’s output, we selected high-risk subjects and, for comparison, included a sample of low-risk individuals following a 2:1 ratio (i.e., for every two high-risk cases, one low-risk control was randomly chosen)

*Revised Results excerpt (line 216-218):* “…121 individuals for serological testing, including 80 classified as high risk for CD and 41 individuals randomly chosen as controls with low risk for CD, following a 2:1 ratio.”

**Results.** Line 187: Why false positives, since several other diseases besides Chagas' disease cause heart problems? Several other diseases present with cardiac dysfunction, and a high percentage of Chagas seropositive individuals do not develop symptoms.

**Reply:** We thank the reviewer for this observation. In this context, we used the term “false positives” to indicate individuals flagged by the ECG-AI as high risk for Chagas disease but who subsequently tested negative on serological assays. To avoid misunderstanding, we have added a clarifying sentence in the Results.

*Revised Results excerpt (around line 225-227):* “They were considered false positives because, although the ECG-AI algorithm suggested a high risk for Chagas disease, their confirmatory serological tests were negative.”

**Results.** Lines 202-203: This affirmation cannot be done because negative samples, in one and/or the commercial tests, were evaluated.

**Reply**: We thank the reviewer for this observation. We agree that the statement could be misleading, as negative samples were not systematically evaluated with the commercial assays. To ensure accuracy and avoid misinterpretation, we have removed this sentence from the Results section in the revised manuscript.

**Conclusions.** It could not be evaluated because of the appointments above described.

**Reply:** We thank the reviewer for this observation. We acknowledge that, in the original version, the Conclusions were affected by the issues raised in other sections. After implementing all suggested modifications and clarifications throughout the manuscript, we have revised the Conclusions accordingly. We respectfully hope that, with these changes, the reviewer will now be able to evaluate the Conclusions in their current form.

**Summary and General Comments.** There are structural issues and a lack of clarity in the text. The methodology is not presented in full, and as a result, there is a lack of information to evaluate the results and discussion presented. The primary objective “estimate the seropositivity rate of *T. cruzi* infection” (lines 89-90) cannot be achieved with the proposal methodology.

**Reply:** We sincerely thank the reviewer for these comprehensive and constructive comments. In response, we carefully revised the manuscript to improve its clarity, structure, and completeness. Specifically, we have expanded the Materials and Methods section to include additional details on the study site (physiogeographic and sociodemographic context based on IBGE data), the structured questionnaire applied, and the laboratory and statistical procedures used. These revisions ensure that the methodology is now fully transparent and reproducible. Regarding the primary objective, we acknowledge that the methodology employed, using AI-ECG risk stratification to select individuals for serological testing, does not allow us to estimate the overall seropositivity rate for *T. cruzi* infection in the general population of Feira de Santana. To address this, we have rephrased the objective and clarified in both the Introduction and Discussion that our study provides an estimate of seropositivity within a subset of individuals identified through a targeted screening strategy, rather than a true population-based prevalence. We also emphasized the need for future community-based surveys to accurately establish population prevalence. We respectfully believe that these modifications address the reviewer’s concerns, strengthen the rigor of the manuscript, and clarify the scope and limitations of our findings.
