# Supplementary material for: Seroepidemiological survey and seropositivity rate for Trypanosoma cruzi infection in a community-based cardiac screening initiative in Feira de Santana, Bahia, Brazil
Source: PLoS Negl Trop Dis. 2026 Jan 2;20(1):e0013892. doi: 10.1371/journal.pntd.0013892 (PMC12782403; doi:10.1371/journal.pntd.0013892)
Supplement: Attachment — Submitted filename: Main document with tracks.docx [file pntd.0013892.s004.docx]

**Seroepidemiological survey and** **seropositivity rate for *Trypanosoma cruzi* infection in a community-based cardiac screening initiative in Feira de Santana, Bahia, Brazil**

**Running title:** Seropositivity rate of *T. cruzi* infection in cardiac screening in Bahia

Felipe Silva Santos de Jesus^1,2¶^, Isabella Moreira Gonzalez Fonseca^3¶^, Ângelo Antônio Oliveira Silva^1,2^, Noilson Lázaro Sousa Gonçalves^1^, Daniel Dias Sampaio^2^, Deborah Bittencourt Mothé Fraga^4^, Paola Alejandra Fiorani Celedon^5^, Dalila Luciola Zanette^5^, Nilson Ivo Tonin Zanchin^6^, Maria Carmo Pereira Nunes^3^, Manoel Otávio da Costa Rocha^3^, Craig Sable^7^, Antônio Luiz Pinho Ribeiro^3^, Fred Luciano Neves Santos^1,2,8*^

^1^ Advanced Health Public Laboratory, Gonçalo Moniz Institute, Oswaldo Cruz Foundation, Salvador, Bahia, Brazil

^2^ Interdisciplinary Research Group in Biotechnology and Epidemiology of Infectious Diseases (GRUPIBE), Gonçalo Moniz Institute, Oswaldo Cruz Foundation, Salvador, Bahia, Brazil

^3^ Medical School and University Hospital, Federal University from Minas Gerais, Belo Horizonte, Minas Gerais, Brazil

^4^Laboratory of Interaction Parasite Host and Epidemiology (LAIPHE), Gonçalo Moniz Institute, Oswaldo Cruz Foundation, Salvador, Bahia, Brazil

^5^Laboratory for Applied Science and Technology in Health, Carlos Chagas Institute, Oswaldo Cruz Foundation, Curitiba, Paraná, Brazil

^6^Structural Biology and Protein Engineering Laboratory, Carlos Chagas Institute, Oswaldo Cruz Foundation, Curitiba, Paraná, Brazil

^7^Ochsner Children’s Hospital, New Orleans, Louisiana, United States

^8^ Integrated Translational Program in Chagas disease from Fiocruz – Fio-Chagas, Rio de Janeiro, Rio de Janeiro, Brazil.

* Corresponding author:

E-mail: fred.santos@fiocruz.br (FNLS)

^¶^These authors contributed equally to this work.

**Abstract**

Chagas disease (CD), caused by *Trypanosoma cruzi*, is a significant public health issue in Latin America, particularly in endemic regions. This study integrates a seroepidemiological survey with large-scale echocardiographic screening conducted in Feira de Santana, a highly endemic city in Bahia, Brazil, to estimate the prevalence of *T. cruzi* infection and identify associated risk factors. Peripheral blood samples were analyzed using in-house ELISA with IBMP chimeric antigens. Among 1,115 participants, 140 underwent serological testing comprising individuals who screened positive based on clinical data, conventional ECG, and ECG-AI, and controls matched in a 2:1 ratio. Of these, 8.5% tested seropositive, with household exposure to triatomines identified as the strongest risk factor (prevalence ratio = 4.38, p = 0.004). Most seropositive individuals were migrants from other endemic areas, underscoring the influence of population mobility on CD epidemiology. This study highlights the importance of integrating diagnostic tools and vector control strategies into community-based health initiatives to improve early detection, reduce disease burden, and inform public health interventions in underserved regions.

**Keywords:** Chagas disease, *Trypanosoma cruzi*, seropositivity rate, risk factors, cardiac screening, vector control.

**Author Summary**

Chagas disease, caused by the parasite *Trypanosoma cruzi*, remains a major health concern in Latin America, especially in areas with limited access to healthcare. In this study, we combined artificial intelligence tools and cardiac screening with serological testing to detect undiagnosed infections in Feira de Santana, a city in the state of Bahia, Brazil. By analyzing electrocardiogram data and environmental risk factors, we identified individuals at risk for Chagas disease and confirmed infection using advanced laboratory tests. Our results showed that people who reported seeing triatomine bugs inside their homes were much more likely to be infected, emphasizing the role of vector exposure in disease transmission. We also found that most infected individuals were unaware of their condition and had migrated from other endemic regions. These findings highlight the importance of combining innovative technologies with community-based screening to improve early diagnosis and guide public health strategies in vulnerable populations.

**Introduction**

Chagas disease (CD), caused by the protozoan *Trypanosoma cruzi*, is a significant public health issue in Latin America, affecting millions and contributing to considerable morbidity and mortality [1,2]. Despite advances in control measures, gaps in early detection and treatment persist, particularly in underserved regions [3]. Chronic CD can lead to cardiac involvement, which represents the leading cause of mortality, highlighting the need for innovative strategies to improve healthcare delivery and patient outcomes.

Bahia State is recognized as one of the Brazilian states with persistent endemicity for Chagas disease, where both vectorial and oral transmission have been historically documented, and recent surveys continue to report significant seroprevalence rates in rural communities [4–6]. The state also harbors the richest biodiversity of triatomine fauna in Brazil, being one of the most infested regions in the country. In Bahia, new species have been described or revalidated, and several triatomine species have shown adaptation to human dwellings, further enhancing the risk of vectorial transmission [7–10]. Within Bahia, Feira de Santana is one of the most populous municipalities and plays a central role as a regional hub. The municipality has reported substantial morbidity and mortality related to CD, reflecting the challenges of disease control and patient management in the state [11]. Socioeconomic disparities and inadequate healthcare infrastructure further exacerbate diagnostic and treatment delays in this region. International initiatives, such as the *Every Heartbeat Matters Program* [12], have emerged as promising approaches to address these challenges. This initiative integrates advanced diagnostic technologies, including telehealth-enabled electrocardiography (teleECG) and targeted echocardiographic screening, to enhance the detection and management of structural heart disease in underserved populations.

This study leverages data from a seroepidemiological survey conducted alongside the *Every Heartbeat Matters* initiative in Feira de Santana. The primary objectives were to estimate the seropositivity rate of *T. cruzi* infection, identify associated risk factors, and assess the feasibility of integrating seroepidemiological surveys with echocardiographic screening programs in endemic settings. The findings aim to inform public health strategies and strengthen the evidence base for effective CD management in high-burden areas.

**Methods**

**Study population**

This cross-sectional study was conducted in Feira de Santana, Bahia, Brazil. Feira de Santana is the second largest city in Bahia, located in the semi-arid region of the Northeast (11° 11′ S, 38° 58′ W) at an altitude of 234 meters, with a tropical savanna climate characterized by a prolonged dry season. According to the Brazilian Institute of Geography and Statistics (IBGE), the municipality has a population of approximately 616,272 inhabitants, making it an important commercial and transport hub in the state [13]. The urban area is characterized by high population density and marked social inequalities, with peripheral neighborhoods presenting limited access to health services, sanitation, and housing infrastructure.

A total of 1,115 adults were recruited for participation in a large-scale screening initiative. All participants completed a structured questionnaire that captured sociodemographic information (age, sex, education level, employment status, household income), environmental and housing conditions (type of dwelling materials, presence of domestic animals, chicken coops, and history of triatomine sightings), family history of Chagas disease, migration background, and previous medical diagnoses. In addition, cardiovascular symptoms were assessed by asking about exercise tolerance, chest pain, syncope, palpitations, and other relevant symptoms. The questionnaire was administered by trained healthcare professionals or supervised medical students at the time of the echocardiographic examination.

All participants underwent electrocardiogram (ECG) acquired with a PC-based system (TEB, São Paulo, Brazil) and transmitted electronically to the telehealth platform, where cardiologists interpreted them and issued standardized reports. ECG abnormalities were classified as major or minor according to the Minnesota Code criteria [14].

Echocardiograms were performed by a team of eight cardiologists using commercially available systems (Vivid Q, GE Healthcare; Affiniti 70, Philips, USA). Images were stored digitally and interpreted immediately after acquisition; when findings were uncertain, two or more cardiologists conducted a consensus read to establish the final diagnosis. Abnormal echocardiograms were defined by the presence of major abnormalities [12].

An ECG-based artificial intelligence (AI) algorithm, implemented as a deep neural network and trained on **standard 12-lead digital ECG waveforms** (time–voltage signals), was used to estimate the risk of positive serology for CD [15]. Based on a combination of epidemiological questions and ECG abnormalities, individuals were categorized into low and high risk for CD. The three selected questions assessed whether participants: (1) had a family member diagnosed with CD, (2) had lived in a region with known triatomine (kissing bug) exposure, and (3) had resided in a wooden house, a known risk factor for vector transmission.

A combination of the answer to these three epidemiological questions and the AI-ECG output was used to generate a risk score, flagging individuals who underwent a serological test for CD. For comparison, controls were randomly selected at a 2:1 ratio (controls to cases) and underwent CD serological testing.

Clinical information was collected using a standardized questionnaire that included sociodemographic characteristics, comorbidities, and history of preexisting heart disease (particularly prior cardiac surgery). Cardiovascular symptoms were assessed by asking participants about exercise tolerance, chest pain, syncope, palpitations, and other relevant manifestations. The questionnaire was administered by trained healthcare professionals or by medical students under direct supervision, at the time of the echocardiogram.

**Specimen Collection and Analysis**

Peripheral blood samples (9 mL) were collected in clot-activator tubes with gel separators (Vacuplast, Brazil). Following a 30-minute clotting period at room temperature, samples were centrifuged at 3,000 × g for 10 minutes. Serum was aliquoted into cryotubes, transported to the Gonçalo Moniz Institute, and stored at −20 °C until analysis.

**Serological Testing**

Anti-*T. cruzi* antibodies were detected using an in-house ELISA based on IBMP chimeric antigens (IBMP-8.1, IBMP-8.2, IBMP-8.3, and IBMP-8.4), following established protocols [16]. Recombinant antigens were expressed in *Escherichia coli* BL21-Star (DE3) and purified via affinity and ion-exchange chromatography. Protein concentrations were quantified fluorometrically using a Qubit 2.0 fluorometer (Invitrogen, Carlsbad, CA, USA). Immunoassays were performed using transparent 96-well flat-bottom microplates (Greiner Bio-One, Austria). The plates were sensitized with the antigens and blocked with a solution of Well-Champion (Ken-Em-Tec Diagnostics A/S, Denmark). Serum samples were diluted 1:100 in PBS-T (PBS containing 0.05% Tween-20; pH 7.4) and incubated at 37 °C for 30 minutes. After washing with PBS-T, a goat anti-human IgG-HRP conjugate (Bio-Manguinhos, FIOCRUZ, Brazil) was added and incubated at 37 °C for 30 minutes. Following additional washing, immune complexes were identified using TBM substrate (Kem-En-Tec, Denmark) with a 10-minute incubation in the dark at room temperature. The reaction was stopped with 0.3 M H₂SO₄, and optical density (OD) was measured at 450 nm using a SPECTRAmax 340PC microplate reader (Molecular Devices, USA).

**Reference Testing**

Latent class analysis (LCA) was employed to classify *T. cruzi* infection status in the absence of a gold standard for chronic CD diagnosis [17–19]. Seropositivity was defined as reactivity in at least two of four ELISAs, with posterior probabilities ranging from 87.9% to 100%. Seronegativity was defined as nonreactivity in at least three ELISAs, with probabilities ranging from 0% to 0.8%. Positive samples were validated using commercial assays, including Gold ELISA Chagas (REM Indústria e Comércio Ltda, Brazil) and Bioelisa Chagas Recombinante (Bioclin Quibasa Química Básica, Brazil).

**Ethics**

This study adhered to the principles of the Declaration of Helsinki and was approved by the Institutional Review Board (IRB) of the Gonçalo Moniz Institute, Oswaldo Cruz Foundation (IGM-FIOCRUZ), Salvador, Bahia, Brazil (protocol no. 67809417.0.0000.0040). Written informed consent was obtained from all participants before enrollment.

**Data Analysis**

Descriptive statistics were used to summarize the demographic and epidemiological characteristics of the study population. Age was reported as the median with the interquartile range (IQR), while categorical variables (e.g., sex, serological test results) were expressed as absolute and relative frequencies. Associations between *T. cruzi* seropositivity and potential risk factors, including prior exposure to triatomines, contact with kissing bugs, presence of a chicken coop, and familial history of CD, were evaluated using contingency tables. Seropositivity rates were calculated as the proportion of seropositive cases among exposed individuals relative to non-exposed individuals, with 95% confidence intervals (CI) and p-values derived from Fisher's exact test. Statistical significance was set at p-value < 0.05. Additionally, prevalence ratio (PR) was performed to assess the association of sociodemographic and environmental factors with positivity. Multiple logistic regression integrated data from 12-lead ECGs plus epidemiological variables to calculate the probability of CD.

**Results**

Between August 24 and 27, 2024, 1,115 individuals participated in the *Every Heartbeat Matters* program, a large-scale ECG and echocardiographic screening, at the Holy House of Mercy in Feira da Santana (Fig 1). In the entire screened population, the median age was 57 years (IQR: 44–64 years), 78.4% were women with a female-to-male ratio of 4.9:1. A total of 284 individuals (27.5%) presented minor ECG abnormalities, while 118 (11.4%) had major abnormalities, according to the Minnesota criteria [14].

Among the selected participants, the AI algorithm identified 121 individuals for serological testing, including 80 classified as high risk for CD and 41 individuals randomly chosen as controls with low risk for CD, following a 2:1 ratio, while 994 were excluded. Of the 73 participants with cardiac abnormalities who underwent blood collection for CD diagnosis, 13 (17.8%) tested positive for *T. cruzi*. In contrast, all 39 control subjects who underwent blood collection tested negative, resulting in a seropositivity rate of 0%. The overall seropositivity rate of *T. cruzi* infection among all tested participants was 11.6% (13/112) (Fig 1).

Among individuals with false-positive AI alerts, ECG abnormalities were observed in six cases classified as major and 16 as minor abnormalities. They were considered false positives because, although the ECG-AI algorithm suggested a high risk for Chagas disease, their confirmatory serological tests were negative. Regarding screening echocardiography, only two participants with confirmed CD exhibited left or right ventricular involvement, while 18 had entirely normal echocardiographic findings.

**Fig 1.** **Study design of a seroepidemiological survey for Chagas disease in Feira de Santana, Bahia, Brazil.** The figure summarizes participant selection within the Every Heartbeat Matters cardiac screening initiative, with ECG and echocardiographic evaluation, risk stratification, and subsequent serological testing using IBMP chimeric antigen–based ELISA and commercial assays. The design follows the Standards for Reporting of Diagnostic Accuracy Studies (STARD) guidelines.

Latent class analysis classified 99 samples (88.4%) as *T. cruzi*-negative, demonstrating no reactivity against the four IBMP antigens, with a posterior probability of zero for positivity. Thirteen samples (11.6%) were classified as *T. cruzi*-positive. Among these, 61.5% (n = 8) reacted with all four IBMP antigens, 15.4% (n = 2) with three antigens, and 23.1% (n = 3) with two antigens. Posterior probabilities for positivity ranged from 96.9% to 100%, confirming their classification as *T. cruzi*-positive, with Gold ELISA Chagas and Bioelisa Chagas Recombinante detecting seropositivity in 100% (13/13) of cases. Table 1 summarizes the serological findings, detailing reactivity patterns across IBMP ELISA assays and commercial kits.

**Table 1.** **Reactivity patterns of serum samples from participants screened for Chagas disease in Feira de Santana, Bahia, Brazil.**

| **Participant number** | **In-house assays with IBMP antigens** | | | | | | **Commercial kits** | |
| --- | --- | --- | --- | --- | --- | --- | --- | --- |
|  | **-8.1** | **-8.2** | **-8.3** | **-8.4** | **LCA** | **P (%) *** | **GEC^1^** | **BIO^2^** |
| 1 | 2.16 | 0.55 | 1.07 | 0.41 | POS | 96.9 | 2.64 | 3.96 |
| 2 | 2.00 | 0.59 | 1.03 | 0.58 | POS | 96.9 | 2.54 | 3.53 |
| 3 | 2.04 | 1.58 | 1.83 | 2.17 | POS | 100 | 12.41 | 3.03 |
| 4 | 0.75 | 1.47 | 1.53 | 1.54 | POS | 100 | 7.35 | 3.22 |
| 5 | 2.75 | 1.84 | 1.72 | 1.85 | POS | 100 | 9.54 | 2.57 |
| 6 | 2.95 | 1.83 | 1.69 | 2.47 | POS | 100 | 7.89 | 3.15 |
| 7 | 2.41 | 1.54 | 1.85 | 1.49 | POS | 100 | 11.01 | 2.99 |
| 8 | 1.44 | 0.62 | 0.58 | 1.01 | POS | 99 | 2.47 | 2.68 |
| 9 | 1.95 | 1.17 | 0.76 | 1.23 | POS | 100 | 5.27 | 2.29 |
| 10 | 1.89 | 1.77 | 1.82 | 2.48 | POS | 100 | 12.71 | 3.57 |
| 11 | 2.84 | 1.08 | 1.90 | 2.03 | POS | 100 | 10.56 | 3.26 |
| 12 | 2.56 | 1.52 | 1.81 | 1.73 | POS | 100 | 10.66 | 2.33 |
| 13 | 2.24 | 1.16 | 1.44 | 1.75 | POS | 100 | 9.01 | 2.61 |

^1^GEC (Gold ELISA Chagas); ^2^BIO (Bioelisa Chagas Recombinante); LCA (Latent Class Analysis); NP (Not Performed); POS (Positive). *Probability of being *T. cruzi*-positive by LCA.

Among *T. cruzi*-positive individuals, the median age was 60 years (IQR: 39–69 years), with a female-to-male ratio of 5.5:1, while *T. cruzi*-negative individuals had a median age of 56 years (IQR: 44–64 years) and a female-to-male ratio of 4.8:1. Notably, 69.2% of seropositive individuals were not born in Feira de Santana (Fig 2), having migrated from other endemic areas in Bahia, including Conceição do Jacuípe (n = 1), Coração de Maria (n = 1), Ipirá (n = 1), Mundo Novo (n = 1), Muniz Ferreira (n = 1), Santa Maria da Vitória (n = 1), Utinga (n = 1), and Santo Estevão (n = 2). This suggests migration as a potential factor influencing local epidemiological trends.

Although most participants were aware of their clinical condition, 23% of *T. cruzi*-positive individuals were unaware of their infection status (Fig 2), highlighting gaps in diagnosis and awareness.

**Fig 2.** **Geographic origins of individuals seropositive for *Trypanosoma cruzi* in Feira de Santana, Bahia, Brazil.** The map shows the municipalities of birth of participants who tested positive for Chagas disease during the Every Heartbeat Matters screening initiative. Public domain digital maps were obtained from the Brazilian Institute of Geography and Statistics (IBGE) and analyzed using QGIS version 3.10 (Geographic Information System, Open-Source Geospatial Foundation Project. http://qgis.osgeo.org).

All *T. cruzi*-positive individuals were older than 46 years and were either retired or beneficiaries of government assistance programs (Fig 2), except for one family cluster. This family, comprising parents and three children, likely contracted the infection through oral transmission after consuming açaí juice in February 2024 near Feira de Santana. At the time of exposure, the parents were 40 and 38 years old. Among the children, a 13-year-old girl and a 9-year-old boy tested positive, while a third child who did not consume the juice tested negative. The infected family members received etiological treatment and remained under clinical supervision.

In univariate analysis, household observation of triatomine bugs showed a higher prevalence of *T. cruzi* seropositivity (PR = 4.38, 95% CI: 1.44–13.3, p = 0.004). However, because this trait was not directly compared between positive and negative individuals in the overall screened population, this finding should be interpreted with caution, as it may reflect a general characteristic of the surveyed community rather than a specific risk factor. Other variables, including general knowledge about the vector, the presence of a chicken coop near the house, and a family history of CD, were not significantly associated with seropositivity. This suggests that while reporting triatomine presence indoors may indicate increased risk, the association requires confirmation in broader community-based analyses (Table 2).

**Table 2.** **Univariate analysis of epidemiological factors associated with *Trypanosoma cruzi* seropositivity in participants from Feira de Santana, Bahia, Brazil.**

| **Variables** | **Univariate analysis** | | |
| --- | --- | --- | --- |
|  | **PR** | **95%CI** | **p-value** |
| Prior exposure to triatomines | 4.38 | 1.44 to 13.3 | 0.004 |
| Presence of a chicken coop | 0.82 | 0.27 to 2.48 | 0.730 |
| Familial history of CD | 1.81 | 0.59 to 5.55 | 0.285 |

PR (Prevalence ratio), β (Log-odds coefficient), CD (Chagas disease) CI (Confidence interval).

**Discussion**

This study leverages a community-based cardiac screening initiative to conduct a seroepidemiological survey, integrating large-scale ECG and echocardiographic screening for the diagnosis of CD. A significant association was identified between the household observation of triatomine bugs and *T. cruzi* seropositivity, underscoring vector exposure as a key factor for CD occurrence in endemic regions. While other environmental factors such as the presence of chicken coops near houses and familial history of CD were not relevant, direct exposure to the vector emerged as the strongest factor associated with higher prevalence of infection. These findings are consistent with previous studies emphasizing the critical role of domestic vector infestation in sustaining transmission cycles [20,21].

The overall seropositivity rate of *T. cruzi* infection in this study (8.5%) is comparable to reports from other endemic areas in Bahia [4–6], reinforcing the persistent public health challenge posed by CD. The high seropositivity rate among individuals reporting the triatomine presence in their households highlights the need for effective vector control programs. In addition to reducing domestic infestation, community education on vector recognition and preventive measures is essential to disrupt transmission dynamics.

A notable observation was that most *T. cruzi*-positive individuals currently residing in Feira de Santana were born in other endemic areas. This suggests that many infections likely occurred before migration, highlighting the impact of population mobility on CD epidemiology. However, it remains unclear whether migration itself serves as an independent risk factor for *T. cruzi* transmission or if these individuals were exposed to their hometowns before relocating. The absence of longitudinal data on the timing of infection limits causal inferences related to migration, as transmission could have occurred before or after resettlement. Furthermore, variations in vector control programs and healthcare access between endemic regions may influence infection risks differently across migrant populations. These findings present efforts to design targeted interventions and emphasize the importance of integrating migrant populations into local healthcare systems. Implementing tailored approaches that improve access to diagnostic and treatment services for migrant communities are essential to reduce the overall burden of CD in endemic regions.

Analysis revealed that individuals who reported finding triatomines in their households presented four times higher prevalence of positivity for *T. cruzi* infection (PR = 4.38, p = 0.004) than individuals who did not report the presence of the vectors inside their houses. This result further underscores the centrality of domestic vector infestation in maintaining transmission cycles and corroborates with previous research identifying in-home triatomine presence as a critical determinant of infection risk [20,21]. The absence of significant associations for other variables, such as general knowledge of the vector or the presence of a chicken coop, reinforces the primacy of direct vector contact in transmission dynamics.

Methodologically, the use of IBMP chimeric antigens for serological screening, complemented by commercial assays for case validation, resulted in high diagnostic accuracy. The application of latent class analysis to classify seropositive cases minimized misclassification bias and enhanced the reliability of our findings. These methodological strengths demonstrate the utility of integrating novel diagnostic tools with community-based health initiatives to effectively address CD in endemic regions.

Although most participants were aware of their clinical condition, it is noteworthy that 23% of individuals identified as *T. cruzi*-positive were unaware of their infection status. This finding highlights the insidious nature of chronic CD, which can remain asymptomatic or manifest with nonspecific symptoms for extended periods, thereby complicating timely diagnosis. Limited access to healthcare services, suboptimal screening practices, and low disease awareness within at-risk communities may contribute to this diagnostic gap. The fact that nearly a quarter of seropositive individuals were previously undiagnosed underscores the need for enhanced community-based screening programs, particularly in endemic areas. By integrating serological testing with cardiac screening initiatives, as demonstrated in our study, early detection and intervention become feasible, potentially curtailing disease progression and reducing long-term morbidity. This observation aligns with earlier research that has documented substantial rates of undiagnosed infections in similar settings, thereby reinforcing the imperative to improve public health strategies aimed at the early identification and management of CD [22–24].

However, the cross-sectional design of this study limits causal inferences, and the relatively small number of seropositive cases may affect the generalizability of our findings. Additionally, the recruitment of participants through a cardiac screening initiative introduces a potential selection bias, as individuals with underlying cardiovascular abnormalities may not be representative of the broader population at risk for *T. cruzi* infection. This could lead to an overestimation of CD seropositivity rate in this specific subgroup while underrepresenting asymptomatic or early-stage infections in the general community. To mitigate sampling bias in serologic testing, we used a computer-generated random selection of controls with low-risk ECG-AI scores to randomly select controls, minimizing bias in serological testing and improving the representativeness of the study sample. Future longitudinal studies with larger, community-based samples, such as those planned within the Oxente Chagas Project [25], are needed to explore the temporal dynamics of infection risk and validate these findings in broader populations.

In conclusion, our study provides critical insights into the epidemiology of *T. cruzi* infection in Feira de Santana, Bahia, emphasizing the central role of vector exposure in CD transmission. The integration of seroepidemiological surveys with cardiac screening initiatives demonstrates a valuable model for identifying at-risk populations and enhancing early detection of CD. Continued investment in vector control, diagnostic innovation, and public health education is essential to reduce the burden of CD in endemic regions [20,26]. These findings underscore the importance of community-based health initiatives in advancing CD control and improving outcomes in underserved and vulnerable populations.

**Acknowledgments**. We are grateful to the Holy House of Mercy team in Feira da Santana for their invaluable assistance in performing this study.

**References**

1.	Laporta GZ, Lima MM, Costa VM, Lima Neto MM, Palmeira SL, Rodovalho SR, et al. Estimation of prevalence of chronic Chagas disease in Brazilian municipalities. Rev Panam Salud Publica. 2024;48: e28. https://doi.org/10.26633/RPSP.2024.28 PMID: 38576844.

2.	Santos EF, Silva ÂAO, Leony LM, Freitas NEM, Daltro RT, Regis-Silva CG, et al. Acute Chagas disease in Brazil from 2001 to 2018: A nationwide spatiotemporal analysis. PLoS Negl Trop Dis. 2020;14(8): e0008445. https://doi.org/10.1371/journal.pntd.0008445 PMID: 32745113.

3.	Chaves GC, Abi-Saab Arrieche M, Rode J, Mechali D, Reis PO, Alves RV, et al. Estimating demand for anti-Chagas drugs: a contribution for access in Latin America. Rev Panam Salud Publica. 2017;41: e45. https://doi.org/10.26633/RPSP.2017.45 PMID: 28614468.

4.	Pavan TBS, Dias DP, Cangussú MM, Dutra VPP, Sampaio DD, Santos FLN. Seroepidemiology of Chagas disease in at-risk individuals in Caraíbas, a city with high endemicity in Bahia State, Brazil. Front Public Health. 2023;11: 1196403. https://doi.org/10.3389/fpubh.2023.1196403 PMID: 37808995.

5.	Escolano P, Liporaci N, Manzan C, Barbosa A, Alves V, Teixeira R, et al. Prevalence of Chagas infection in Catolândia-Bahia. Rev Soc Bras Med Trop. 1989;22(3): 159–60. https://doi.org/10.1590/s0037-86821989000300009 PMID: 2518611.

6.	Aras R, Gomes I, Veiga M, Melo A. Vectorial transmission of Chagas' disease in Mulungu do Morro, Northeastern of Brazil. Rev Soc Bras Med Trop. 2003;36(3): 359–63. https://doi.org/10.1590/s0037-86822003000300008 PMID: 12908037.

7.	Ribeiro-Jr G, Araújo RF, Carvalho CMM, Cunha GM, Lanza FC, Miranda DLP, et al. Triatomine fauna in the state of Bahia, Brazil: What changed after 40 years of the vector-control program? Rev Soc Bras Med Trop. 2022;55: e07322021. https://doi.org/10.1590/0037-8682-0732-2021 PMID: 35894404.

8.	Costa J, Dale C, Galvão C, Almeida CE, Dujardin JP. Do the new triatomine species pose new challenges or strategies for monitoring Chagas disease? An overview from 1979-2021. Mem Inst Oswaldo Cruz. 2021;116: e210015. https://doi.org/10.1590/0074-02760210015 PMID: 34076075.

9.	Gurgel-Gonçalves R, Galvão C, Costa J, Peterson AT. Geographic distribution of chagas disease vectors in Brazil based on ecological niche modeling. J Trop Med. 2012;2012: 705326. https://doi.org/10.1155/2012/705326 PMID: 22523500.

10.	Costa J, Almeida CE, Dotson EM, Lins A, Vinhaes M, Silveira AC, et al. The epidemiologic importance of Triatoma brasiliensis as a Chagas disease vector in Brazil: A revision of domiciliary captures during 1993-1999. Mem Inst Oswaldo Cruz. 2003;98(4): 443–9. https://doi.org/10.1590/S0074-02762003000400002 PMID: 12937751.

11.	Carvalho CMM, Ribeiro-Jr G, Gurgel-Gonçalves R, Andrade LS, Moraes CA, Figueiredo MAA. Spatio-temporal trends in mortality due to Chagas disease in the State of Bahia, Brazil, from 2008 to 2018. Rev Soc Bras Med Trop. 2024;57: e004172024. https://doi.org/10.1590/0037-8682-0058-2024 PMID: 39476074.

12.	Diamantino AC, Nascimento BR, Nunes MCP, Sable CA, Oliveira KKB, Rabelo LC, et al. Impact of incorporating echocardiographic screening into a clinical prediction model to optimise utilisation of echocardiography in primary care. Int J Clin Pract. 2021;75(3): e13686. https://doi.org/10.1111/ijcp.13686 PMID: 32852108.

13.	Instituto Brasileiro de Geografia e Estatística (IBGE). Feira de Santana. In: IBGE Cidades [Internet]. 2023 [cited 25 Sep 2025]. Available: https://cidades.ibge.gov.br/brasil/ba/feira-de-santana/panorama.

14.	Ribeiro ALP, Marcolino MS, Prineas RJ, Lima-Costa MF. Electrocardiographic abnormalities in elderly Chagas disease patients: 10-year follow-up of the Bambui Cohort Study of Aging. J Am Heart Assoc. 2014;3(1): e000632. https://doi.org/10.1161/JAHA.113.000632 PMID: 24510116.

15.	Jidling C, Gedon D, Schön TB, Oliveira CDL, Cardoso CS, Ferreira AM, et al. Screening for Chagas disease from the electrocardiogram using a deep neural network. PLoS Negl Trop Dis. 2023;17(7): e0011118. https://doi.org/10.1371/journal.pntd.0011118 PMID: 37399207.

16.	Santos FLN, Celedon PAF, Zanchin NIT, Brasil TAC, Foti L, Souza WV, et al. Performance assessment of four chimeric Trypanosoma cruzi antigens based on antigen-antibody detection for diagnosis of chronic Chagas disease. PLoS One. 2016;11(8): e0161100. https://doi.org/10.1371/journal.pone.0161100 PMID: 27517281.

17.	Santos FLN, Campos ACP, Amorim LDAF, Silva ED, Zanchin NIT, Celedon PAF, et al. Highly accurate chimeric proteins for the serological diagnosis of chronic Chagas disease: A latent class analysis. Am J Trop Med Hyg. 2018;99(5): 1174–9. https://doi.org/10.4269/ajtmh.17-0727 PMID: 30226130.

18.	Santos EF, Leony LM, Silva ÂAO, Daltro RT, Freitas NEM, Vasconcelos LCM, et al. Assessment of Liaison XL Murex Chagas diagnostic performance in blood screening for Chagas disease using a reference array of chimeric antigens. Transfusion. 2021;61(9): 2701–9. https://doi.org/10.1111/trf.16583 PMID: 34240750.

19.	Santos EF, Silva ÂAO, Freitas NEM, Almeida MCC, Araújo FLV, Celedon PAF, et al. Performance of chimeric Trypanosoma cruzi antigens in serological screening for Chagas disease in blood banks. Front Med. 2022;9: 852864. https://doi.org/10.3389/fmed.2022.852864 PMID: 35330587.

20.	Dias JCP, Ramos AN, Gontijo ED, Luquetti A, Shikanai-Yasuda MA, Coura JR, et al. 2 nd Brazilian consensus on Chagas disease, 2015. Rev Soc Bras Med Trop. 2016;49(1_Suppl): 3–60. https://doi.org/10.1590/0037-8682-0505-2016 PMID: 27982292.

21.	Pan American Health Organization (PAHO). Actualización de la estimación de la enfermedad de Chagas en los países endémicos de las Américas 2018. In: PAHO [Internet]. 17 Jun 2025 [cited 26 Sep 2025]. Washington, D.C. Available: https://iris.paho.org/handle/10665.2/67559.

22.	Fidalgo ASOBV, Costa AC, Ramos Júnior AN, Leal LKAM, Martins AMC, Filho JDS, et al. Seroprevalence and risk factors of Chagas disease in a rural population of the Quixeré municipality, Ceará, Brazil. Rev Soc Bras Med Trop. 2021;54: e0247-2020. https://doi.org/10.1590/0037-8682-0247-2020 PMID: 33681912.

23.	Freitas EC, Oliveira MF, Vasconcelos ASOB, Filho JDS, Viana CEM, Gomes KCMS, et al. Analysis of the seroprevalence of and factors associated with Chagas disease in an endemic area in Northeastern Brazil. Rev Soc Bras Med Trop. 2017;50(1): 44–51. https://doi.org/10.1590/0037-8682-0242-2016 PMID: 28327801.

24.	Santana MA, Ferreira ALS, Santos LVB, Campos JHF, Sena LLJ, Mendonça VJ. Seroprevalence of Chagas disease in rural communities at Campinas do Piauí city, Brazil. Trop Med Int Health. 2021;26(3): 281–9. https://doi.org/10.1111/tmi.13516 PMID: 33159709.

25.	Santos FLN, Pavan TBS, Valle CS, Sampaio DD, Vasconcelos LCM, Cristóbal MH, et al. The Oxente Chagas Bahia Project: Evaluating the efficacy of a rapid diagnostic test and treatments for Chagas disease. Mem Inst Oswaldo Cruz. 2024;119: e240140. https://doi.org/10.1590/0074-02760240140 PMID: 39476029.

26.	Santos FLN, Costa VM, Silva RA. Chagas disease in Brazil: New challenges and perspectives for old problems. Mem Inst Oswaldo Cruz. 2025;120: e240279. https://doi.org/10.1590/0074-02760240279 PMID: 40699036.
